# Endogenous aldehyde-induced DNA–protein crosslinks are resolved by transcription-coupled repair

**DOI:** 10.1038/s41556-024-01401-2

**Published:** 2024-04-10

**Authors:** Yasuyoshi Oka, Yuka Nakazawa, Mayuko Shimada, Tomoo Ogi

**Affiliations:** 1 Department of Genetics, Research Institute of Environmental Medicine (RIeM), Nagoya University, Nagoya, Japan; 2 Department of Human Genetics and Molecular Biology, Nagoya University Graduate School of Medicine, Nagoya, Japan; 3 Division of Animal Medical Science, Center for One Medicine Innovative Translational Research (COMIT), Nagoya University, Nagoya, Japan; 4 Division of Molecular Physiology and Dynamics, Institute for Glyco-core Research (iGCORE), Nagoya University, Nagoya, Japan

**Keywords:** Transcriptional regulatory elements, Nucleotide excision repair

## Abstract

DNA–protein crosslinks (DPCs) induced by aldehydes interfere with replication and transcription. Hereditary deficiencies in DPC repair and aldehyde clearance processes cause progeria, including Ruijs–Aalfs syndrome (RJALS) and AMeD syndrome (AMeDS) in humans. Although the elimination of DPC during replication has been well established, how cells overcome DPC lesions in transcription remains elusive. Here we show that endogenous aldehyde-induced DPC roadblocks are efficiently resolved by transcription-coupled repair (TCR). We develop a high-throughput sequencing technique to measure the genome-wide distribution of DPCs (DPC-seq). Using proteomics and DPC-seq, we demonstrate that the conventional TCR complex as well as VCP/p97 and the proteasome are required for the removal of formaldehyde-induced DPCs. TFIIS-dependent cleavage of RNAPII transcripts protects against transcription obstacles. Finally, a mouse model lacking both aldehyde clearance and TCR confirms endogenous DPC accumulation in actively transcribed regions. Collectively, our data provide evidence that transcription-coupled DPC repair (TC-DPCR) as well as aldehyde clearance are crucial for protecting against metabolic genotoxin, thus explaining the molecular pathogenesis of AMeDS and other disorders associated with defects in TCR, such as Cockayne syndrome.

## Abstract (web summary)

Three studies identify a transcription-coupled DNA–protein crosslink repair pathway that depends on the Cockayne syndrome proteins and the proteasome.

## Main

The maintenance and faithful spatiotemporal expression of genetic information is crucial for life^1–3^. Among genotoxic stresses, endogenous reactive aldehydes induce covalent adducts and crosslinks with biomolecules, which subsequently interfere with replication and transcription^4^. To counteract the toxicity of aldehydes, organisms have developed systems to eliminate them and repair the damage they cause. Formaldehyde is a common one-carbon (1C) metabolite generated from various cellular processes that occurs at tens-of-micromolar concentrations in the human body^5–7^. Formaldehyde is primarily detoxified by the gene products of *ADH5* (alcohol dehydrogenase 5), which encodes a glutathione-dependent formaldehyde dehydrogenase, and *ALDH2* (aldehyde dehydrogenase 2)^8,9^. Simultaneous loss of function in these two genes causes a multisystem disorder called AMeD syndrome (AMeDS), which is characterized by bone marrow failure (aplastic anaemia), intellectual disabilities (mental retardation) and developmental defects (dwarfism) due to the accumulation of endogenous formaldehyde and the overload of DNA repair pathways with formaldehyde-derived DNA damage^10,11^.

The most common forms of DNA damage induced by aldehydes are DNA-interstrand crosslinks (ICLs) and DNA–protein crosslinks (DPCs). These types of damage are particularly deleterious because they interfere with replication and cause cell death when left unrepaired^12,13^. ICL repair relies on the FANC genes, which, when mutated, cause a bone marrow failure syndrome known as Fanconi anaemia (FA)^14^. This mechanism, also referred to as the FA pathway, is activated during S-phase and removes ICLs through sequential processes^15^. The importance of the FA pathway in coping with aldehyde-induced DNA damage is further underscored by the fact that loss of either of the FANC genes, along with defects in aldehyde metabolic processes, leads to the development of more severe phenotypes in mice and humans^16–18^. In parallel with ICLs, both enzymatic- and aldehyde-induced DPCs are also predominantly eliminated during DNA replication through the involvement of a metalloprotease, SPRTN, which is compromised in cases of cancer-predisposed Ruijs–Aalfs progeroid syndrome (RJALS)^19–22^. The ubiquitin–proteasome system also plays a role in the proteolysis of DPCs^23–25^. After the initial degradation of DPC, the excision of remnant peptides, as well as DPCs of smaller sizes (<11 kDa), may involve nucleotide excision repair (NER)^26,27^.

Aldehyde-induced ICLs and DPCs also block RNA synthesis. However, how they are resolved during transcription is less clear. Intriguingly, patients with AMeDS display the combined features of FA and Cockayne syndrome (CS)^28,29^, the latter being a progressive neurodegenerative disease associated with transcription-coupled repair (TCR) deficiency, suggesting the involvement of a missing DNA repair pathway for removing aldehyde-induced damage from active genes. The TCR process, a concept where DNA lesions located in transcribed regions are promptly removed compared to other locations, is initiated by the stalling of an elongating RNA polymerase II (RNAPII) at DNA damage^1–3^. The conventional mechanism involves processing of the stalled RNAPII by the ubiquitination of RPB1—the largest RNAPII subunit—at the lysine-1268 residue by the CSA and CSB (Cockayne syndrome A and B) proteins. RNAPII ubiquitination triggers its interaction with the UVSSA (ultraviolet-stimulated scaffold protein A) protein, followed by UVSSA–lysine-414 ubiquitination to facilitate the recruitment of transcription factor II H (TFIIH)^30,31^. TFIIH unwinds nucleotides surrounding the DNA lesion and further recruits the XPA (xeroderma pigmentosum complementation group A) protein and endonucleases (XPF/ERCC1 and XPG) to remove ~30-bp damage-containing nucleotides. Eventually, DNA polymerases and ligases fill and seal the residual gap to complete the repair. Yet, it remains unclear whether the conventional TCR process can remove DNA lesions beyond those addressed by the TC-NER mechanism.

In this Article we report that aldehyde-induced transcription-blocking lesions are efficiently repaired by TCR. Using a newly developed technique to measure the genome-wide distribution of DPCs, we reveal that the removal of DPCs in transcribed regions requires conventional TCR factors and the proteasome. We also demonstrate that a mouse model lacking both aldehyde clearance processes and the TCR pathway exhibits aggravated AMeDS manifestations, represented by severe haematopoietic abnormalities and systemic asthenia. Collectively, the removal of DPCs is pivotal in protecting against aldehyde-induced transcription roadblocks, and we provide mechanistic insights into the pathogenesis of diseases associated with defects in aldehyde clearance and TCR.

## Results

### Formaldehyde-induced DPCs are removed from active genes

**Fig. 1:**
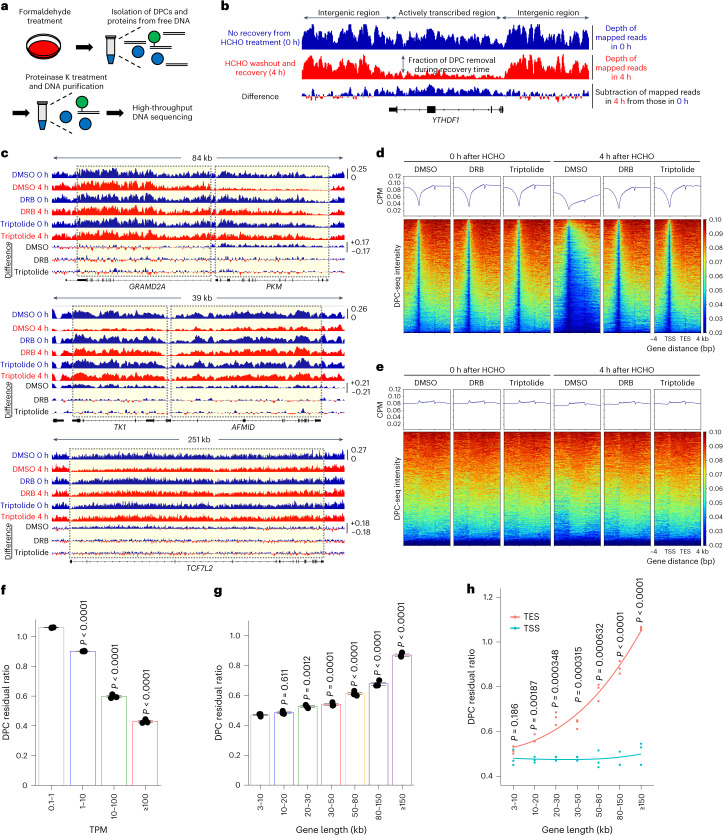
Formaldehyde-induced DPCs are removed from active genes. **a**, Schematic representation of the experimental procedure for DPC-seq in cultured cells. **b**,**c**, Genome browser snapshot showing DPC-seq signals for HeLa cells treated with formaldehyde (HCHO) (**b**), and in the presence of DMSO, DRB or triptolide (**c**). **d**,**e**, Metagene profile and heatmap from DPC-seq within or near transcribed regions of genes with TPM ≥ 30 (**d**) and 0.1 ≤ TPM < 1 (**e**). CPM, counts per million mapped reads; TSS, transcription start site; TES, transcription end site. Data represent the average of three replicates. **f**, DPC residual ratios represent the proportions of sequence coverage of each gene in cells recovered for 4 h divided by that of cells not recovered after formaldehyde treatment, shown in four TPM bins. **g**, DPC residual ratios, shown in seven gene-length bins. Means (±s.e.m.) from three independent experiments are shown. Statistical significance was evaluated with Dunnett’s multiple comparison test. **h**, DPC residual ratios on regions including 1 kb downstream of the TSS and regions including 1 kb upstream of the TES, shown in seven gene-length bins. Means (±s.e.m.) from three independent experiments are shown. Two-sided unpaired *t*-test. Source numerical data are available as source data.

**Extended Data Fig. 1:**
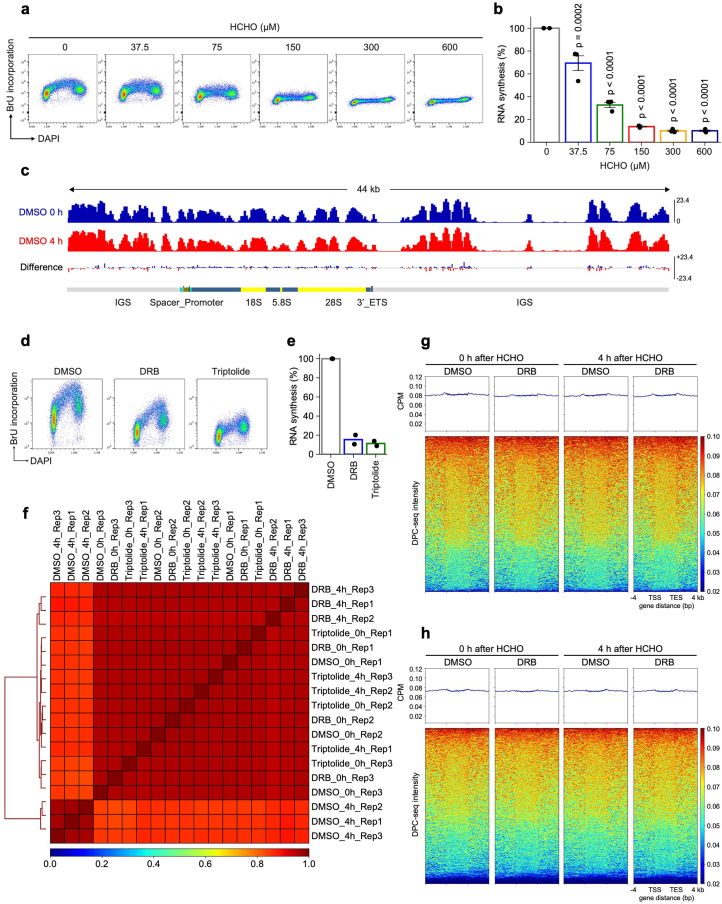
Formaldehyde treatment blocks transcription. (**a**) 5′-bromo-uridine (BrU) incorporation in HeLa cells treated with various concentration of formaldehyde (HCHO) was measured by FACS analysis. Data represent the three independent experiments. (**b**) Quantification of data in (a). Means (±s.e.m.) from three independent experiments are shown. Statistical significance was evaluated with Dunnett’s multiple comparison test. (**c**) Genome browser snapshot showing DPC-seq signals for HeLa cells treated with formaldehyde within a ribosomal DNA unit. IGS: intergenic spacer; ETS: external transcribed spacer. (**d**) BrU incorporation in HeLa cells treated with DMSO, DRB or triptolide was measured by FACS analysis. Data represent the two independent experiments. (**e**) Quantification of data in (**d**). Means from two independent experiments are shown. (**f**) A hierarchically clustered heatmap showing Spearman’ rank correlation coefficients of DPC-seq signal intensities (CPM) between three replicates in HeLa cells treated with DMSO, DRB or triptolide. (**g** and **h**) Metagene profile and heatmap from input samples within and near transcribed regions of genes with TPM ≥ 30 (**g**) and 0.1 ≤ TPM < 1 (h). TSS: transcription start site; TES: transcription end site. n = 1. Source numerical data are available in source data.

We initially measured the effects of formaldehyde on transcription. HeLa cells were treated with formaldehyde for 1 h and labelled with 5′-bromo-uridine (BrU) to monitor nascent RNA synthesis. The level of transcription decreased after formaldehyde treatment in a dose-dependent manner (Extended Data Fig. 1a,b). Because formaldehyde causes a range of types of macromolecular damage that interfere with transcription, we further focused on the effects of formaldehyde-induced DPCs. To investigate the repair kinetics of DPCs in transcribed regions, we developed a next-generation sequencing (NGS) method called DPC-seq, which enables genome-wide mapping of DPCs through a combination of protein-conjugated DNA precipitation and NGS library preparation. Briefly, cells exposed to formaldehyde were lysed in sodium dodecyl sulfate (SDS)-denaturing buffer, followed by DNA shearing and KCl-SDS precipitation^32^. After digestion of the DNA-conjugated proteins with proteinase K, the DNA samples were purified and subjected to high-throughput sequencing (Fig. 1a). Cells were exposed to 600 μM formaldehyde for 1 h, followed by 0 h and 4 h of recovery time after a formaldehyde washout before DPC detection. At 4 h post treatment, we observed a marked reduction of DPC-derived high-throughput sequence reads across the gene bodies, suggesting an efficient removal of DPCs from transcribed regions (Fig. 1b shows a representative gene, *YTHDF1*). DPCs were efficiently removed from the *PKM* gene, which is actively transcribed (transcripts per kilobase per million (TPM) = 1,730), whereas removal was not apparent in an inactive gene, *GRAMD2A* (TPM = 0.3), which is transcribed in the opposite direction (Fig. 1c). Ribosomal DNA (rDNA) repeats are transcribed by RNA polymerase I (RNAPI)^33^, and we did not observe any overt removal of DPCs from a single rDNA unit in the 4-h recovery period (Extended Data Fig. 1c). Furthermore, DPC removal from representative active genes (*PKM*, *TK1*, *AFMID* and *TCF7L2*) was suppressed in cells treated with RNAPII transcription inhibitors, 5,6-dichlorobenzimidazole1-β-d-ribofuranoside (DRB) or triptolide; this was highly reproducible in different experiments (Fig. 1c and Extended Data Fig. 1d–f). Consistent with these observations in individual genes, aggregation plots depict similar profiles of DPC removal across the genome (Fig. 1d,e and Extended Data Fig. 1g,h). Collectively, these data indicate that formaldehyde-induced DPCs are exclusively eliminated from active genes associated with RNAPII transcription activity.

To further study the effects of transcript levels and the size of genes on DPC repair, we reanalysed DPC removal profiles in genes classified according to their transcriptional activity and length. Consistent with the effects of transcription inhibitors, we observed a negative correlation between the DPC residual ratios 4 h post treatment with formaldehyde and the expression levels of genes (Fig. 1f). Notably, we found that removal of DPCs was delayed for genes that were ≥20 kb in length compared to genes with lengths of 3–10 kb (Fig. 1g). We also compared DPC residual ratios between nearby transcription start sites (TSS; including 1 kb downstream of a TSS) and transcription end sites (TES; including 1 kb upstream of a TES). As expected, we observed equally efficient removal of DPCs both from the TSSs and TESs of shorter genes, whereas DPCs in TESs were removed more slowly than those in TSSs in longer genes (Fig. 1h).

### DPC repair by the conventional TCR pathway

**Fig. 2:**
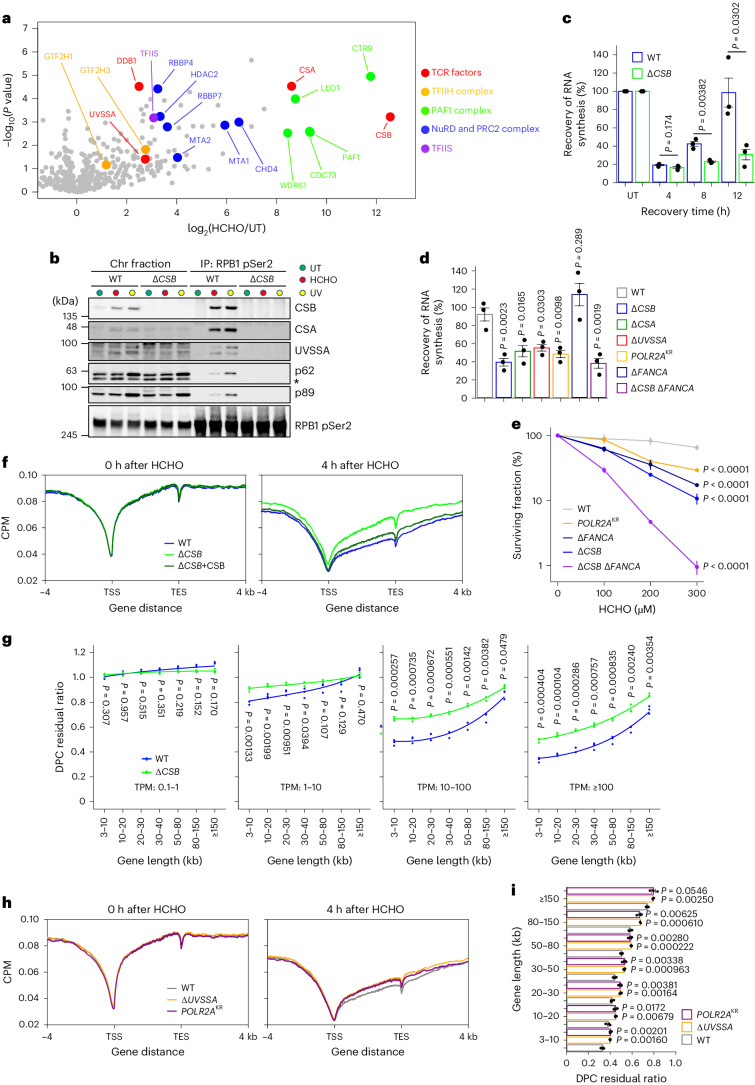
DPC repair by the TCR pathway. **a**, Volcano plot of MS analyses illustrating the formaldehyde-induced protein interactions with elongating RNAPII. The plot displays the log_2_-fold change and significance (−log_10_(*P* value)) assessed by an unpaired two-sided *t*-test: permutation-based FDR < 0.05 (*n* = 3). Supplementary Table 1 provides the full results. **b**, Chromatin fractions of HeLa cells after formaldehyde treatment or UV irradiation were subjected to immunoprecipitation with anti-RPB1 CTD phospho-Ser2 antibodies, followed by immunoblotting with the indicated antibodies. The asterisk indicates non-specific bands. Experiments were independently replicated twice with consistent results. **c**, RRS after formaldehyde treatment in WT and Δ*CSB* HeLa cells. Cells were treated with 1,200 μM HCHO for 1 h, followed by 12-h incubation for recovery. Quantification of BrU incorporation is shown (means ± s.e.m.; *n* = 3 independent experiments). Two-sided unpaired *t*-test. **d**, RRS after formaldehyde treatment in the TCR- or FA- pathway-deficient HeLa cell lines. Cells were treated with 1,750 μM HCHO for 1 h, followed by 18-h incubation for recovery. The quantification of ethynyluridine (EU) incorporation is shown (means ± s.e.m.; *n* = 3 independent experiments). Dunnett’s multiple comparison test. **e**, Clonogenic survival of HeLa cell lines exposed to various doses of formaldehyde. Results from three independent experiments (mean ± s.e.m.) are shown. Dunnett’s multiple comparison test. **f**, Metagene profile from DPC-seq within or near transcribed regions of genes with TPM ≥ 30 at 0 and 4 h after formaldehyde washout in WT, Δ*CSB* and Δ*CSB* stably expressing CSB-WT HeLa cells. Data represent the average of three replicates. **g**, DPC residual ratios in WT or Δ*CSB* HeLa cells, shown in seven gene-length bins with TPM ≥ 100. Means (±s.e.m.) from three independent experiments are shown. Two-sided unpaired *t*-test. **h**, Metagene profile from DPC-seq within or near transcribed regions of genes with TPM ≥ 30 at 0 and 4 h after formaldehyde washout in WT, Δ*UVSSA* and *POLR2A*^KR^ cells. Data represent the average of three replicates. **i**, DPC residual ratios in WT, Δ*UVSSA* and *POLR2A*^KR^ cells are shown in seven gene-length bins with TPM ≥ 100. Means (±s.e.m.) from three independent experiments are shown. Two-sided unpaired *t*-test. Source numerical data and unprocessed blots are available in source data. Source data

**Extended Data Fig. 2:**
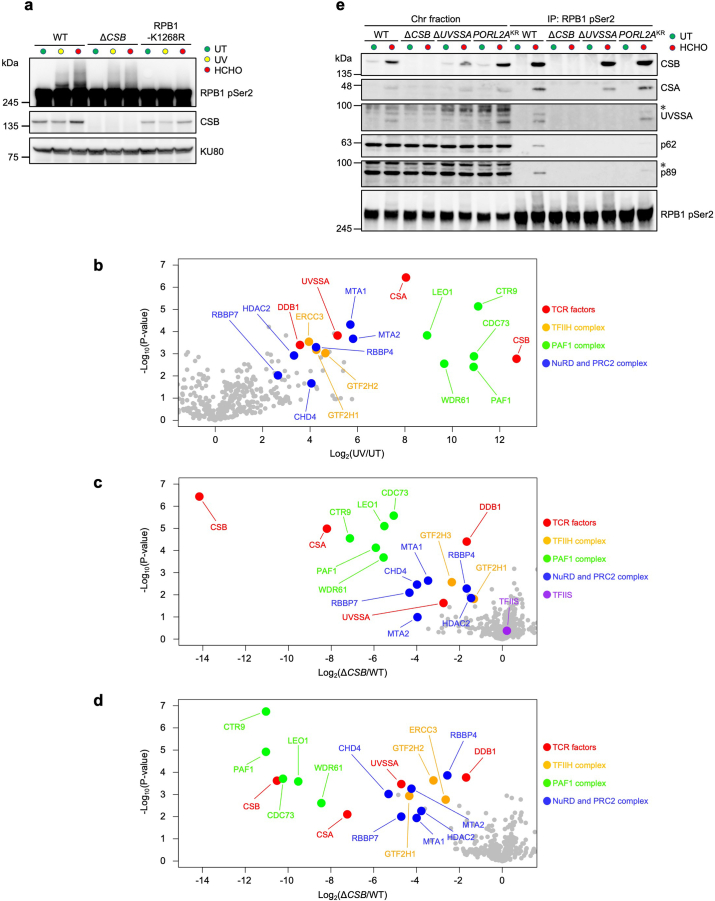
CSB-dependent ubiquitination signaling is activated in response to formaldehyde treatment. (**a**) Whole cell extracts of WT, Δ*CSB* and *POLR2A*^KR^ HeLa cells after formaldehyde treatment or UV irradiation were analysed by immunoblotting with indicated antibodies. Experiments were independently replicated twice with consistent results. (**b**) Volcano plot of mass spectrometry analyses illustrating the UV-induced interaction of elongating RNAPII. Chromatin fractions of HeLa WT cells after UV irradiation (UV) or untreated (UT) were subjected to immunoprecipitation with anti-RPB1 CTD phospho-Ser2 antibodies followed by mass spectrometric analysis. The plot displays the log2 fold change and the significance (-log10(P-value)) assessed by unpaired two-sided t test: permutation-based FDR < 0.05 (n = 3). Selected prospective elongating RNAPII interactors are highlighted. See Supplementary Table 4 for full results. (**c**) Volcano plot of mass spectrometry analyses illustrating the CSB-dependent formaldehyde-induced interaction of elongating RNAPII. Chromatin fractions of HeLa WT and Δ*CSB* cells after formaldehyde treatment were subjected to immunoprecipitation with anti-RPB1 CTD phospho-Ser2 antibodies followed by mass spectrometric analysis. The plot displays the log2 fold change and the significance (-log10(P-value)) assessed by unpaired two-sided t test: permutation-based FDR < 0.05 (n = 3). Selected prospective elongating RNAPII interactors are highlighted. See Supplementary Table 5 for full results. (**d**) Volcano plot of mass spectrometry analyses illustrating the CSB-dependent UV-induced interaction of elongating RNAPII. Chromatin fractions of HeLa WT and Δ*CSB* cells after UV irradiation were subjected to immunoprecipitation with anti-RPB1 CTD phospho-Ser2 antibodies followed by mass spectrometric analysis. The plot displays the log2 fold change and the significance (-log10(P-value)) assessed by a permutation-based *t*-test (n = 3). Selected prospective elongating RNAPII interactors are highlighted. See Supplementary Table 6 for full results. (**e**) Chromatin fractions of WT, Δ*CSB*, Δ*UVSSA* and *POLR2A*^KR^ HeLa cells after formaldehyde treatment were subjected to immunoprecipitation with anti-RPB1 phospho-Ser2 antibodies followed by immunoblotting with indicated antibodies. * indicates non-specific bands. Experiments were independently replicated twice with consistent results. Unprocessed blots are available in source data. Source data

In a previous report, genome-scale CRISPR screens against various DNA-damaging reagents identified an interaction between the loss of TCR and sensitivity to formaldehyde^34^. Moreover, neurodegeneration and kidney failure were observed in mice lacking both *Adh5* and a TCR gene, *Csb*^35^. Although these reports suggest that the repair of aldehyde-derived DNA damage may involve TCR, the mechanistic insights into the repair of this damage in transcribed regions remain obscure. We therefore started to overview the effects of RNAPII stalling caused by formaldehyde-induced transcription roadblocks. To initially identify factors interacting with RNAPII in a formaldehyde-specific manner, we performed a proteome analysis by immunoprecipitating elongating RNAPII (phosphorylated RPB1, at the C-terminal domain, Ser2) followed by mass spectrometry (MS). The proteome analysis detected the RNAPII interaction with TCR initiation factors (red, Fig. 2a), including CSB, CSA and UVSSA, as well as TFIIH- (yellow, Fig. 2a), PAF1- (green, Fig. 2a) and PRC- (blue, Fig. 2a) complexes, in a formaldehyde-specific manner. We noted that elongating RNAPII was ubiquitinated at the RPB1-K1268 residue dependently on CSB following formaldehyde treatment (Extended Data Fig. 2a). Based on the similarity of observations after UV irradiation^31^, the ubiquitination of RPB1 with K48- and K63-linked chains may occur to facilitate the recruitment of conventional TCR factors in response to aldehyde-induced DNA damage. Consistent with this, we identified the recruitment of TCR initiation factors to RNAPII as being CSB-dependent following formaldehyde treatment, using MS and immunoblotting (Fig. 2b and Extended Data Fig. 2b–d)^30,31,36^. Additionally, recruitment of the TFIIH core complex is also dependent on UVSSA and RPB1-K1268 ubiquitination, as well as CSB (Extended Data Fig. 2e).

**Extended Data Fig. 3:**
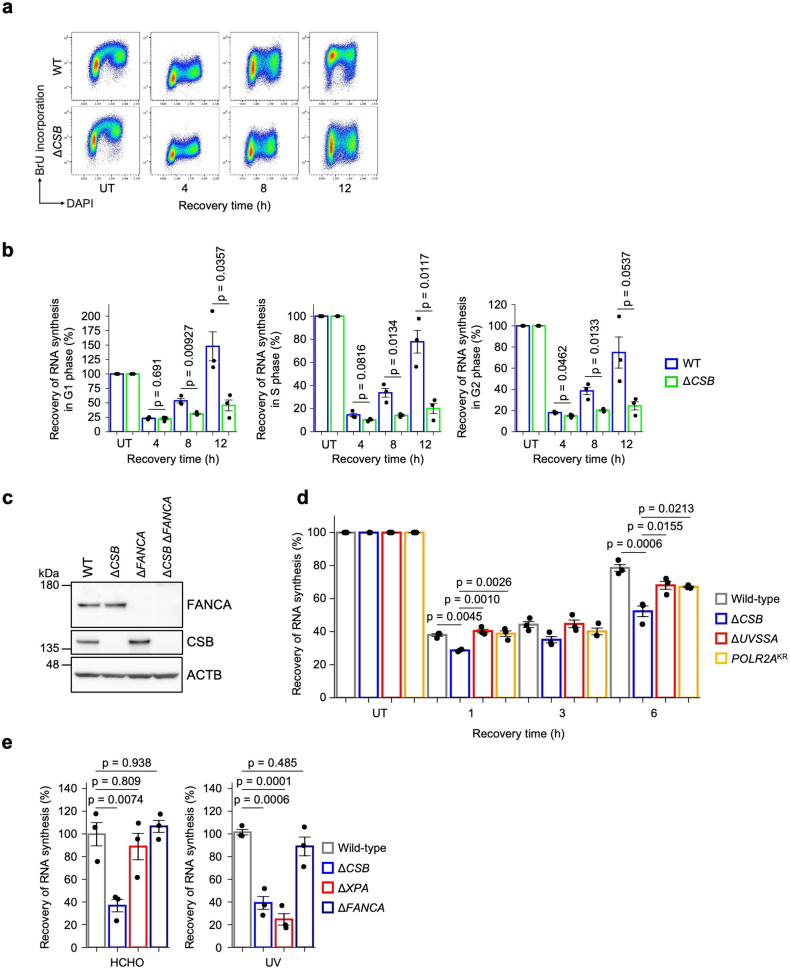
CSB-dependent TCR initiation signaling is required for RRS. (**a**) RRS after formaldehyde treatment in WT and Δ*CSB* cells. BrU incorporation in HeLa WT and Δ*CSB* cells treated with formaldehyde was measured by FACS analysis. Data represent the three independent experiments. (**b**) Quantification of BrU incorporation in G1, S and G2 phase is shown (means ± s.e.m.; n = 3 independent experiments). Two-sided unpaired *t*-test. (**c**) Cell extracts from HeLa WT, Δ*CSB*, Δ*FANCA* and Δ*CSB* Δ*FANCA* cells were analysed by immunoblotting with indicated antibodies. Experiments were independently replicated twice with consistent results. (**d**) RRS after formaldehyde treatment in HeLa WT, Δ*CSB*, Δ*UVSSA*, *POLR2A*^KR^ cells. Cells were treated with 500 μM HCHO for 1 h followed by recovered at the indicated time points. Quantification of 5-ethynyluridine (EU) incorporation is shown (means ± s.e.m.; n = 3 independent experiments). Statistical significance was evaluated with Tukey–Kramer multiple comparison test. (**e**) RRS after formaldehyde treatment or UV irradiation in HeLa WT, Δ*CSB*, Δ*XPA* and Δ*FANCA* cells. Cells were treated with 1750 μM HCHO for 1 h followed by 18 h incubation, or irradiated with 6 J/m^2^ UV followed by 12 h incubation for RNA synthesis recovery. Quantification of EU incorporation is shown (means ± s.e.m.; n = 3 independent experiments). Statistical significance was evaluated with Dunnett’s multiple comparison test. Source numerical data and unprocessed blots are available in source data. Source data

Because TCR is indispensable for prompt transcription recovery after exposure to various genotoxic insults, we measured the recovery of RNA synthesis (RRS) after formaldehyde treatment. Consistently, TCR-deficient Δ*CSB* cells exhibited delayed RRS, irrespective of cell cycle, post-formaldehyde treatment (Fig. 2c and Extended Data Fig. 3a,b). Similar to the conventional TC-NER process in response to UV damage, Δ*CSA* and Δ*UVSSA* cells, along with RPB1-K1268R (*POLR2A*^KR^) cells lacking the major damage-dependent ubiquitination site, also exhibited defects in RRS following formaldehyde treatment. Remarkably, Δ*FANCA* cells compromised in the FA core complex display no overt RRS deficiency (Fig. 2d and Extended Data Fig. 3c). The RRS deficiency observed in Δ*CSB* cells was more evident at earlier time points compared to Δ*UVSSA* or *POLR2A*^KR^ cells, indicating that transcription recovery following formaldehyde treatment relies more on CSB than on the RPB1-K1268 ubiquitination and UVSSA (Extended Data Fig. 3d). Unexpectedly, we did not observe any RRS defects after treatment with formaldehyde in Δ*XPA* cells (Extended Data Fig. 3e). Either the loss of *FANCA* or *CSB* or the ubiquitination of RPB1 sensitized cells to formaldehyde, and this effect was even more pronounced in Δ*CSB* and Δ*FANCA* double-deficient cells (Fig. 2e and Extended Data Fig. 3c). These data suggest that the FA core complex is dispensable for transcription recovery in cells exposed to formaldehyde, so the FA pathway may be a parallel DNA repair mechanism that targets formaldehyde-induced DNA lesions independently of TCR.

**Extended Data Fig. 4:**
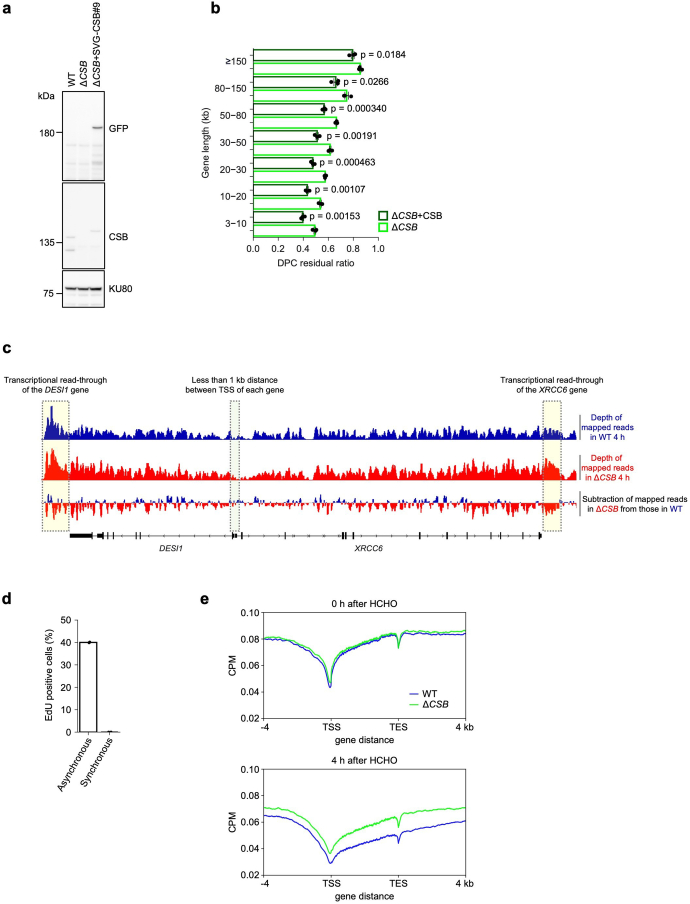
CSB-dependent DPC repair. (**a**) Extracts of HeLa WT, Δ*CSB* and Δ*CSB* stably expressing Strep-V5-EGFP (SVG)-CSB-WT were analysed by immunoblotting with indicated antibodies. Experiments were independently replicated twice with consistent results. (**b**) DPC residual ratios in HeLa Δ*CSB* and Δ*CSB* stably expressing CSB-WT cells are shown in seven gene length bins with TPM ≥ 100. Means (±s.e.m.) from three independent experiments are shown. Two-sided unpaired *t*-test. (**c**) Genome browser snapshot showing DPC-seq signals for HeLa WT and Δ*CSB* cells treated with formaldehyde. (**d**) EdU incorporation in HeLa cells synchronised by a double thymidine followed by nocodazole block and release protocol. Data represent the two independent experiments. (**e**) Metagene profile from DPC-seq within or near transcribed regions of genes with TPM ≥ 30 at 0 and 4 h after formaldehyde washout in WT and Δ*CSB* HeLa cells synchronised by a double thymidine followed by nocodazole block and release protocol. Data represent the average of three replicates. Source numerical data and unprocessed blots are available in source data. Source data

To further obtain direct evidence for the involvement of TCR factors in DPC removal, we performed DPC-seq in wild-type and Δ*CSB* HeLa cells. In response to formaldehyde exposure, the loss of CSB caused a significant reduction in DPC repair activity in highly transcribed regions of the genome, which was restored by the ectopic expression of CSB-WT in Δ*CSB* cells (Fig. 2f and Extended Data Fig. 4a,b). The differences in DPC repair kinetics were particularly prominent in highly expressed and shorter genes (Fig. 2g). We noticed that the DPC depth of coverage, even outside gene bodies, was also affected in Δ*CSB* cells, showing slower repair kinetics compared to wild-type cells. This effect is probably attributed to bidirectional transcription and read-through contributing to the rapid removal of DNA lesions located in those regions (Extended Data Fig. 4c). We then investigated whether DNA replication affects the removal of DPCs. To this end, we performed DPC-seq in wild-type and Δ*CSB* cells synchronized at the G1/S-boundary by a protocol involving double thymidine followed by nocodazole block and release. The results revealed that DPCs were removed in actively transcribed regions in G1-phase, similar to asynchronous cells, and the process also depended on CSB (Extended Data Fig. 4d,e). Moreover, similar trends (although not pronounced) in the delay of DPC repair in transcriptionally active genes were observed in Δ*UVSSA* and *POLR2A*^KR^ cells (Fig. 2h,i). Thus, we conclude that conventional TCR factors have an important role in DPC repair after treatment with formaldehyde.

### DPC removal by VCP/p97 and the proteasome in active genes

**Fig. 3:**
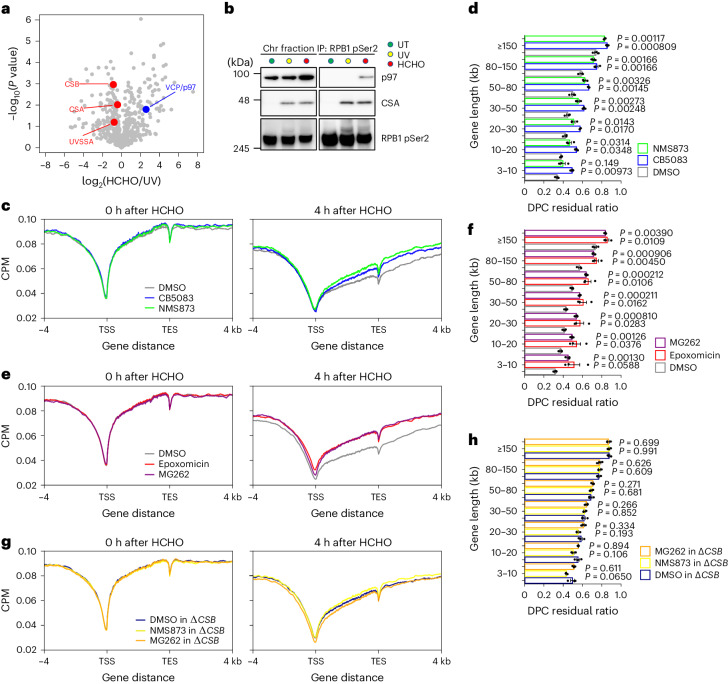
DPC removal by VCP/p97 and the proteasome in active genes. **a**, Volcano plot of MS analyses illustrating the interacting proteins with elongating RNAPII following formaldehyde treatment compared to UV irradiation. The plot displays the log_2_(fold change) and significance (−log_10_(*P* value)). Unpaired two-sided *t*-test. Permutation-based FDR < 0.05 (*n* = 3). Supplementary Table 2 provides the full results. **b**, Chromatin fractions of HeLa cells after formaldehyde treatment or UV irradiation were subjected to immunoprecipitation with anti-RPB1 CTD phospho-Ser2 antibodies, followed by immunoblotting with the indicated antibodies. Experiments were independently replicated twice with consistent results. **c**, Metagene profile from DPC-seq within or near transcribed regions of genes with TPM ≥ 30 at 0 and 4 h after formaldehyde washout in cells treated with VCP/p97 inhibitors. Data represent the average of two replicates. **d**, DPC residual ratios in cells treated with VCP/p97 inhibitors, shown in seven gene-length bins with TPM ≥ 100. Means (±s.e.m.) from three independent experiments are shown. Two-sided unpaired *t*-test. **e**, Metagene profile from DPC-seq within or near transcribed regions of genes with TPM ≥ 30 at 0 and 4 h after formaldehyde washout in HeLa cells treated with proteasome inhibitors. Data represent the average of three replicates. **f**, DPC residual ratios in cells treated with proteasome inhibitors are shown in seven gene-length bins with TPM ≥ 100. Means (±s.e.m.) from three independent experiments are shown. Two-sided unpaired *t*-test. **g**, Metagene profile from DPC-seq within or near transcribed regions of genes with TPM ≥ 30 at 0 and 4 h after formaldehyde washout in Δ*CSB* HeLa cells treated with the indicated inhibitors. Data represent the average of three replicates. **h**, DPC residual ratios in Δ*CSB* HeLa cells treated with the indicated inhibitors, shown in seven gene-length bins with TPM ≥ 100. Means (±s.e.m.) from three independent experiments are shown. Two-sided unpaired *t*-test. Source numerical data and unprocessed blots are available in the source data. Source data

To identify specific factors involved in repairing aldehyde-induced DNA damage through the TCR pathway, we performed a comparative interactome analysis following UV irradiation and formaldehyde treatment. Elongating RNAPII was immunoprecipitated after DNA-damaging treatment and subjected to MS analysis. This enabled us to find the ‘ATP-driven chaperone valosin-containing protein, (VCP)/p97’ as a formaldehyde-specific interacting partner of elongating RNAPII (Fig. 3a). The interaction was further verified by immunoblotting (Fig. 3b). In the process of DPC elimination, proteolytic degradation of covalently bound proteins is required before the incision processes. VCP/p97, with its ATPase activity, binds to DPC and extracts it to deliver the unfolded protein to the proteasome^37^. To further study the roles of VCP/p97 in DPC removal from transcribed regions, we performed DPC-seq following treatment of cells with the VCP/p97 inhibitors CB5083 and NMS873, observing a pronounced inhibition of DPC removal (Fig. 3c,d). Additionally, we also examined the impact of proteasomal degradation on the removal of DPCs. Treatment with the proteasome inhibitors epoxomicin and MG262 significantly impeded DPC removal kinetics, as measured by DPC-seq after formaldehyde treatment (Fig. 3e,f).

**Extended Data Fig. 5:**
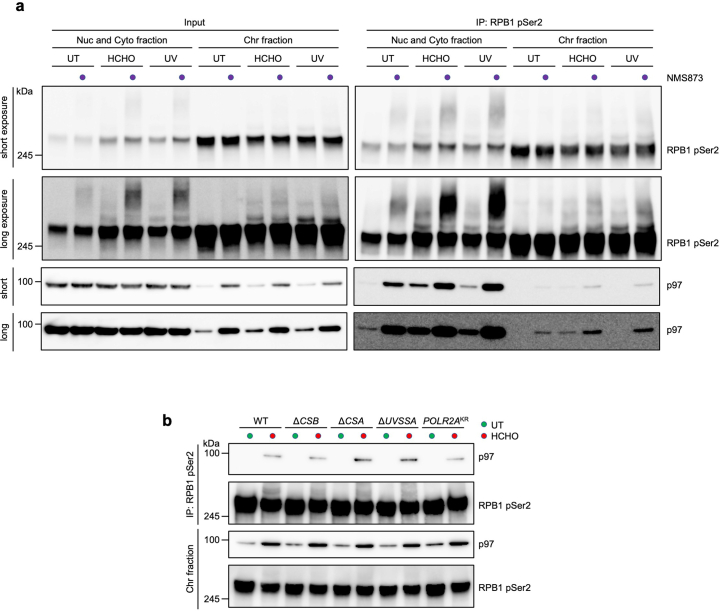
The role of VCP/p97 in response to formaldehyde-induced DNA damage. (**a**) HeLa cell extracts fractionated into nuclear/cytoplasm (Nuc/Cyto) and chromatin after formaldehyde treatment or UV irradiation were subjected to immunoprecipitation with anti-RPB1 CTD phospho-Ser2 antibodies followed by immunoblotting with indicated antibodies (n = 1). (**b**) Chromatin fractions of HeLa WT, Δ*CSB*, Δ*CSA*, Δ*UVSSA* and *POLR2A*^KR^ cells after formaldehyde treatment or UV irradiation were subjected to immunoprecipitation with anti-RPB1 CTD phospho-Ser2 antibodies followed by immunoblotting with indicated antibodies. Experiments were independently replicated twice with consistent results. Unprocessed blots are available in source data. Source data

Given that DNA damage-stalled RNAPII is ubiquitinated by CSB, such modified RNAPII becomes a potential target of VCP/p97 for degradation. To address this point, we examined the degradation profiles of RNAPII in response to DNA damage in combination with p97 inhibition (Extended Data Fig. 5a). Cells were fractionated to separately examine degradation in the soluble nuclear/cytoplasm (Nuc/Cyto) fraction as well as in the chromatin fraction. We observed an accumulation of ubiquitinated RNAPII in the soluble fraction in cells treated with NMS873 following formaldehyde treatment or UV irradiation. This suggests that a substantial amount of elongating RNAPII undergoes ubiquitination and degradation during DNA damage-induced global transcription shutdown. In contrast, chromatin-bound ubiquitinated RNAPII, representing RNAPII stalling at DNA damage, remained mostly unchanged with or without NMS873 treatment. This indicates that these chromatin-bound ubiquitinated RNAPII are not subjected to degradation nor targeted by VCP/p97, irrespective of the types of DNA damage.

Unlike UV irradiation, formaldehyde treatment resulted in a stable RNAPII-p97 interaction in the chromatin fraction, even without NMS873 treatment. However, this interaction was much weaker than that observed for the degradation of ubiquitinated RNAPII in the soluble fraction following DNA damage. Considering that the recruitment of p97 to stalled RNAPII is not affected in Δ*CSB* or *POLR2A*^KR^ cells (Extended Data Fig. 5b), this interaction is independent of the conventional TCR process regulated by CSB and ubiquitination of RNAPII at the RPB1-K1268 residue.

In parallel, we evaluated DPC removal kinetics by DPC-seq in Δ*CSB* cells treated with NMS873 or MG262. The inhibitors exhibited no additive effects on the removal of DPC from actively transcribed regions in Δ*CSB* cells, indicating that the inhibition of DPC removal is epistatic to Δ*CSB* (Fig. 3g,h). Collectively, these data suggest that the unfolding and degradation of DPC by VCP/p97 and the proteasome are required for efficient TCR of formaldehyde-induced DPC damage, which operate in the same pathway as CSB.

### Transcription-coupled removal of histone-DPC

**Fig. 4:**
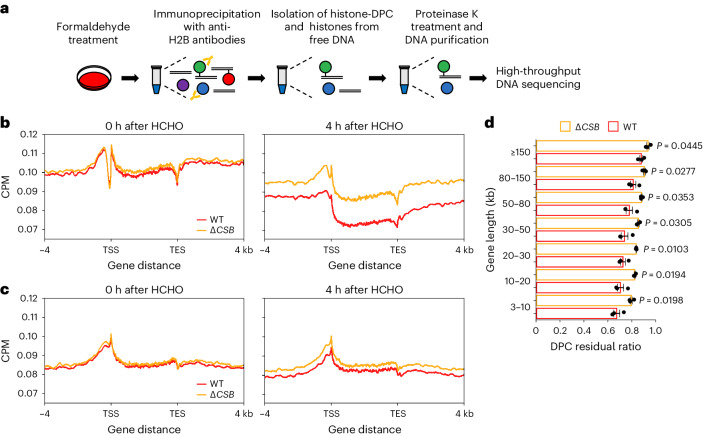
Transcription-coupled removal of histone-DPC. **a**, Schematic representation of the experimental procedure for histone-DPC-seq in cultured cells. **b**,**c**, Metagene profile from histone-DPC-seq within or near transcribed regions of genes with TPM ≥ 30 (**b**) and 0.1 ≤ TPM < 1 (**c**) at 0 and 4 h after formaldehyde washout in WT and Δ*CSB* HeLa cells. Data represent the average of three replicates. **d**, Histone-DPC residual ratios in WT or Δ*CSB* HeLa cells, shown in seven gene-length bins with TPM ≥ 100. Means (±s.e.m.) from three independent experiments are shown. Two-sided unpaired *t*-test. Source numerical data are available in the source data.

A single nucleosome is composed of ~147 bp of DNA wrapped around a histone octamer, which consists of two copies of each core histone (H2A, H2B, H3 and H4)^38^. As they always associate with DNA, histones are highly possible candidate DPCs that block RNAPII progression^39,40^. To investigate whether histone-DPCs are targets of the TCR pathway, we developed an optional DPC-seq procedure to detect DNA-histone conjugates and measure their distribution. We performed immunoprecipitation of histones using anti-H2B antibodies followed by standard DPC-seq (Fig. 4a). We observed a sharp decline in DPC signals nearby TSSs 0 h after treatment with formaldehyde in highly expressed genes, possibly due to the presence of abundant naked DNA (Fig. 4b). This observation is consistent with nucleosome eviction in transcriptionally active promoter regions^41,42^. However, this decline in signals was not detectable in inactive genes (Fig. 4c). Although histone-DPCs were efficiently removed from actively transcribed regions of the genome at 4 h after formaldehyde treatment, the loss of CSB significantly delayed removal (Fig. 4b,d). Similar to the findings on entire DPC removal (Fig. 2f), delays in histone-DPC repair in Δ*CSB* cells were also more pronounced in shorter genes (Fig. 4d). In total, CSB-dependent TCR targets nucleosomes crosslinked with DNA.

### TFIIS protects against aldehyde-induced transcription stress

**Fig. 5:**
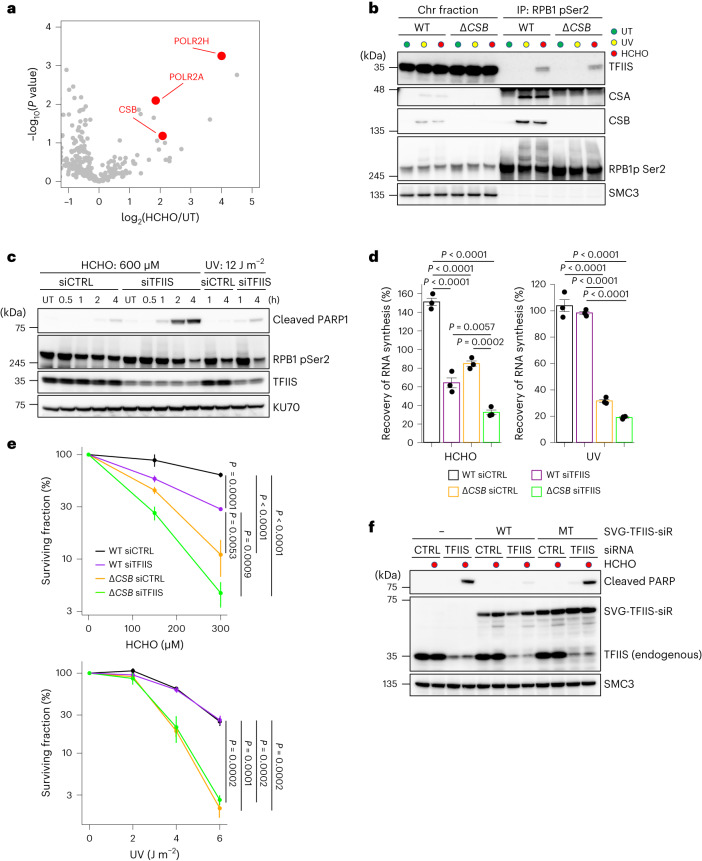
TFIIS protects against aldehyde-induced transcription stress. **a**, Volcano plot of MS analyses illustrating the formaldehyde-induced protein interactions with TFIIS. The plot displays log_2_(fold change) and significance (−log_10_(*P* value)) assessed by unpaired two-sided *t*-test. Permutation-based FDR < 0.05 (*n* = 3). Supplementary Table 3 provides the full results. **b**, Chromatin fractions of WT and Δ*CSB* HeLa cells after formaldehyde treatment or UV irradiation were subjected to immunoprecipitation with anti-RPB1 CTD phospho-Ser2 antibodies, followed by immunoblotting with the indicated antibodies. Experiments were independently replicated twice with consistent results. **c**, HeLa cells transfected with non-targeting control (siCTRL) or TFIIS siRNAs (siTFIIS) were treated with formaldehyde or UV irradiation. Cell extracts were analysed by immunoblotting with the indicated antibodies. Experiments were independently replicated twice with consistent results. **d**, RRS after formaldehyde treatment or UV irradiation in WT and Δ*CSB* HeLa cells transfected with siCTRL or siTFIIS. Cells were treated with 1,000 μM HCHO for 1 h or irradiated with 8 J m^−2^ UV, followed by 13 h of incubation for recovery. Quantification of EU incorporation is shown (means ± s.e.m.; *n* = 3 independent experiments). Statistical significance was evaluated with a Tukey–Kramer multiple comparison test. **e**, Clonogenic survival of WT and Δ*CSB* HeLa cells transfected with siCTRL or siTFIIS exposed to various doses of formaldehyde or UV light. Results from three independent experiments (mean ± s.e.m.) are shown. Statistical significance was evaluated with a Tukey–Kramer multiple comparison test. **f**, HeLa cells stably expressing TFIIS-WT or TFIIS-MT transfected with the indicated siRNAs were treated or not with formaldehyde. Cell extracts were analysed by immunoblotting with the indicated antibodies. Experiments were independently replicated twice with consistent results. Source numerical data and unprocessed blots are available in the source data. Source data

The formaldehyde-induced RNAPII interactome analysis shown in Fig. 2a initially identified the transcription factor IIS (TFIIS, encoded by *TCEA1*), which promotes cleavage of RNA transcripts by binding to the backtracked RNAPII, allowing it to resume transcription^43^. Reverse interactome analyses using antibodies against TFIIS confirmed the interactions between TFIIS and RNAPII, as well as CSB, after formaldehyde treatment (Fig. 5a), suggesting that TFIIS is recruited to RNAPII stalled at aldehyde-induced DNA damage. Intriguingly, the interaction between elongating RNAPII and TFIIS was exclusively detected after treatment with formaldehyde, demonstrating a specific response to formaldehyde-induced DNA damage, unlike after UV irradiation. Furthermore, the loss of CSB did not affect this interaction (Fig. 5b).

**Extended Data Fig. 6:**
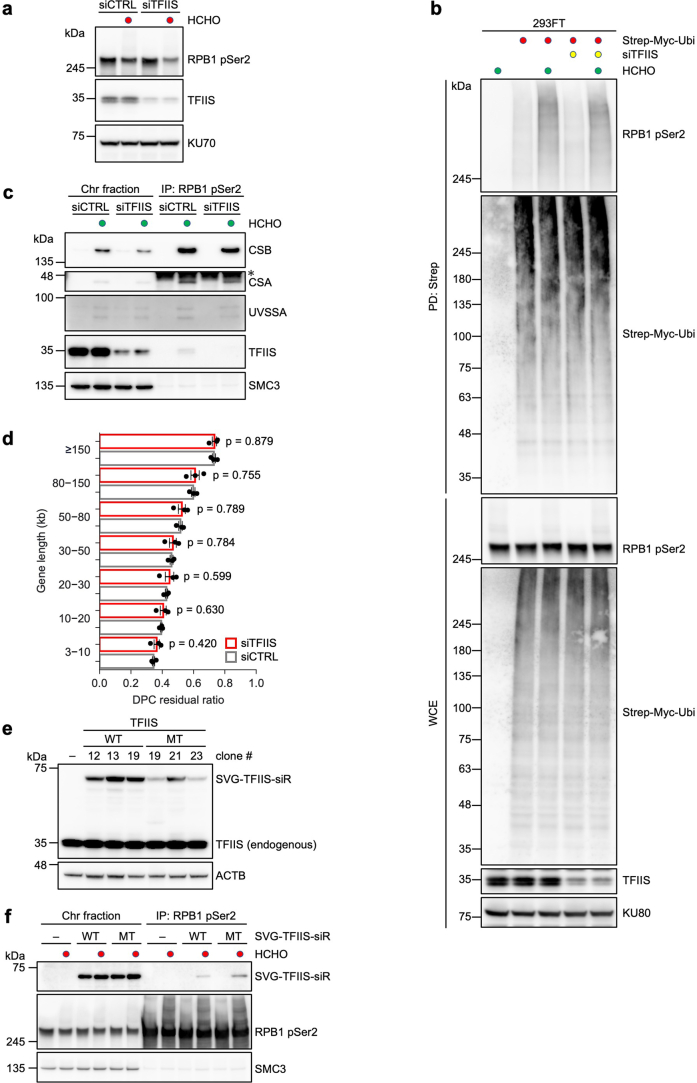
TFIIS is required for safeguard against formaldehyde-induced transcription stress. (**a**) Whole cell extracts of HeLa WT cells transfected with non-targeting control (siCTRL) or TFIIS siRNAs (siTFIIS) were treated with formaldehyde. Cell extracts were analysed by immunoblotting with indicated antibodies. Experiments were independently replicated twice with consistent results. (**b**) 293FT cells transfected with siCTRL or siTFIIS were transfected with Strep-Myc-Ubiquitin (Ubi). Whole cell extracts were subjected to Strep-Tactin pull-down followed by immunoblotting with indicated antibodies. Experiments were independently replicated twice with consistent results. (**c**) Chromatin fractions of HeLa cells transfected with siCTRL or siTFIIS were subjected to immunoprecipitation with anti-RPB1 CTD phospho-Ser2 antibodies followed by immunoblotting with indicated antibodies (n = 1). * indicates non-specific bands. (**d**) DPC residual ratios in HeLa WT cells transfected with siCTRL or siTFIIS are shown in seven gene length bins with TPM ≥ 100. Means (±s.e.m.) from three independent experiments are shown. Two-sided unpaired *t*-test. (**e**) Whole cell extracts of HeLa cells stably expressing TFIIS-WT or TFIIS-MT (D282A and E283A) were analysed by immunoblotting with indicated antibodies (n = 1). (**f**) Chromatin fractions of HeLa cells stably expressing TFIIS-WT or TFIIS-MT treated or not with formaldehyde were subjected to immunoprecipitation with anti-RPB1 CTD phospho-Ser2 antibodies followed by immunoblotting with indicated antibodies. Experiments were independently replicated twice with consistent results. Source numerical data and unprocessed blots are available in source data. Source data

To gain further insight into the role of TFIIS in response to formaldehyde DNA damage, we selectively depleted its expression using siRNAs. Subsequently, cleaved PARP1, a marker for apoptosis, was detected exclusively in TFIIS-depleted cells after formaldehyde treatment, underscoring the specific involvement of TFIIS in the apoptotic response to formaldehyde-induced DNA damage, distinct from the response to UV irradiation (Fig. 5c). Furthermore, the loss of TFIIS function caused defects in RRS and sensitized cells to formaldehyde, with an even more pronounced effect observed in cells with double-depletion of TFIIS and CSB (Fig. 5d,e). Notably, TFIIS depletion did not exhibit any sensitivity to UV irradiation (Fig. 5e). We next analysed the potential role of TFIIS in the ubiquitination and degradation of DNA damage-stalled RNAPII and DPC repair following formaldehyde treatment. We observed no apparent effects on the levels and ubiquitination of elongating RNAPII in TFIIS-depleted cells after formaldehyde treatment (Extended Data Fig. 6a,b). Moreover, there were no overt defects in the recruitment of TCR factors at stalled RNAPII and DPC repair in TFIIS-depleted cells (Extended Data Fig. 6c,d).

Amino acid substitutions at the TFIIS D282 and E283 residues, which are located in the acidic loop of TFIIS, lose the ability of transcript cleavage by RNAPII, resulting in increased levels of arrested RNAPII, a slow transcription elongation rate, and genome instability under unperturbed conditions in human cells^44,45^. To study the importance of transcript cleavage by RNAPII in response to formaldehyde-induced DNA damage, we generated cell lines stably expressing siRNA-resistant wild-type (WT, clone #12) and mutant forms (MT, D282A and E283A, clone #21) of TFIIS (Extended Data Fig. 6e). Although this TFIIS mutant may have dominant-negative effects on cell proliferation^44,45^, we did not observe any overt cellular growth defects in cells stably expressing TFIIS-MT, which is probably due to its mild expression levels. Although the mutations did not affect the interaction between TFIIS and elongating RNAPII following formaldehyde exposure (Extended Data Fig. 6f), the sensitivity measured by apoptotic induction was not rescued in cells expressing TFIIS-MT, whereas cells expressing wild-type TFIIS showed a substantial suppression (Fig. 5f).

Collectively, these findings imply that TFIIS may act on RNAPII stalled by DPC to facilitate the resumption of transcription through its ability to cleave transcripts when the removal of DPCs is completed, which was a previously unrecognized primary role of TFIIS.

### TCR deficiency exacerbates phenotypes of AMeDS model mice

**Extended Data Fig. 7:**
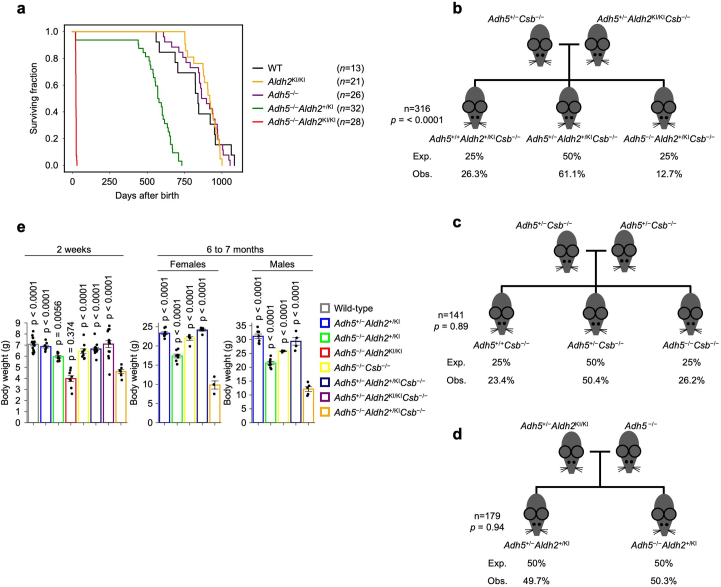
Loss of *Csb* exacerbates the phenotypes of aldehyde metabolism-deficient AMeDS mice. (**a**) Kaplan-Meier curves with log-rank test (partially adapted from Oka et al.^11^) show a significant decrease in survival of *Adh5*^−/−^*Aldh2*^+/KI^ or *Adh5*^−/−^*Aldh2*^KI/KI^ AMeDS animal compared to WT mice (p < 0.0001 or p < 0.0001, respectively). (**b**) Observed and expected frequencies of mice at 2 weeks of age from intercrossed *Adh5*^+/−^*Csb*^−/−^ mice with *Adh5*^+/−^*Aldh2*^KI/KI^*Csb*^−/−^ mice. One-sided chi-square test shows significant difference between observed and expected (p < 0.0001). (**c**) Observed and expected frequencies of mice at 2 weeks of age from intercrossed between *Adh5*^+/−^*Csb*^−/−^ mice. One-sided chi-square test shows no significant difference between observed and expected (p = 0.89). (**d**) Observed and expected frequencies of mice at 2 weeks of age from intercrossed of *Adh5*^−/−^ mice with *Adh5*^+/−^*Aldh2*^KI/KI^ mice. Chi-square test shows no significant difference between observed and expected (p = 0.94). (**e**) Body weights of individual mice at 2 weeks of age (means ± s.e.m.; n = 18 for wild-type, n = 7 for *Adh5*^*+*/−^*Aldh2*^*+*/KI^, n = 8 for *Adh5*^−/−^*Aldh2*^+/KI^, n = 8 for *Adh5*^−/−^*Aldh2*^KI/KI^, n = 9 for *Adh5*^−/−^*Csb*^−/−^, n = 12 for *Adh5*^*+*/−^*Aldh2*^*+*/KI^*Csb*^−/−^, n = 14 for *Adh5*^*+*/−^*Aldh2*^KI/KI^*Csb*^−/−^, n = 6 for *Adh5*^−/−^*Aldh2*^+/KI^*Csb*^−/−^), female mice at 6 to 7 months of age (means ± s.e.m.; n = 8 for *Adh5*^*+*/−^*Aldh2*^*+*/KI^, n = 7 for *Adh5*^−/−^*Aldh2*^+/KI^, n = 5 for *Adh5*^−/−^*Csb*^−/−^, n = 5 for *Adh5*^*+*/−^*Aldh2*^*+*/KI^*Csb*^−/−^, n = 3 for *Adh5*^−/−^*Aldh2*^+/KI^*Csb*^−/−^), and male mice at 6 to 7 months of age (means ± s.e.m.; n = 7 for *Adh5*^*+*/−^*Aldh2*^*+*/KI^, n = 8 for *Adh5*^−/−^*Aldh2*^+/KI^, n = 4 for *Adh5*^−/−^*Csb*^−/−^, n = 4 for *Adh5*^*+*/−^*Aldh2*^*+*/KI^*Csb*^−/−^, n = 5 for *Adh5*^−/−^*Aldh2*^+/KI^*Csb*^−/−^). Statistical significance was evaluated with Dunnett’s multiple comparison test. *P* compared to *Adh5*^−/−^*Aldh2*^+/KI^*Csb*^−/−^ mice. Source numerical data are available in source data.

We have recently reported that a functional single-nucleotide polymorphism, *ALDH2* rs671 (c.1510G>A, p.E504K), which is known to cause the Asian alcohol flushing phenotype, in combination with biallelic loss-of-function mutations in *ADH5* leads to AMeDS, a digenic multisystem disorder^10,11^. Our mouse model of AMeDS, harbouring double mutations (*Adh5*^−/−^ and *Aldh2*-E506K, equivalent to the human *ALDH2*-E504K and hereafter called *Aldh2*^KI^) recapitulates the major clinical findings of AMeDS, such as short life span, growth failure and haematopoietic abnormalities (Extended Data Fig. 7a)^11^. The cells of patients with AMeDS are hyper-sensitive to formaldehyde, and mice lacking both Adh5 and Aldh2 enzymatic activities show increased levels of formaldehyde in blood, indicating that the ADH5 and ALDH2 function to detoxify endogenous formaldehyde in a coordinated manner^10,11^. The evidence suggests that the loss of functions of *Adh5* and *Aldh2* increase the levels of aldehyde-induced DPCs, leading to transcriptional stress in mice.

**Fig. 6:**
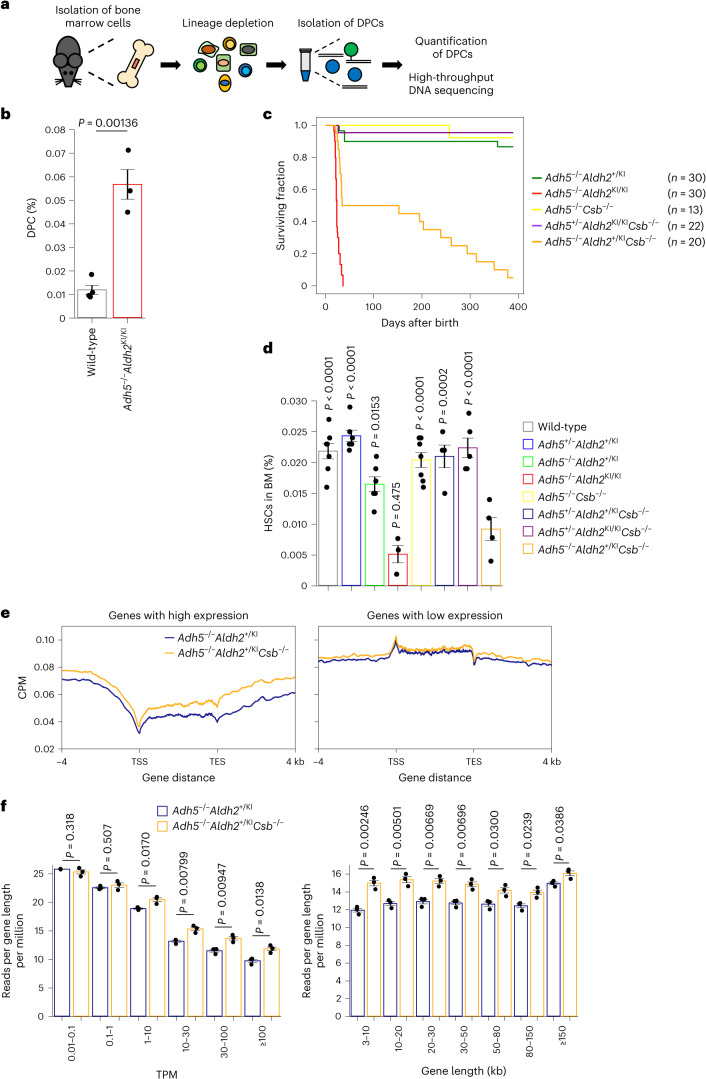
TCR is required for the removal of DPCs in mice. **a**, In vivo DPC-seq: schematic representation of the experimental procedure for the measurement of endogenous aldehyde-induced DPCs in mice. **b**, Quantification of DPCs in lineage-depleted bone marrow cells from WT and AMeDS model mice (means ± s.e.m.; *n* = 4 for wild-type, *n* = 3 for *Adh5*^−/−^*Aldh2*^KI/KI^). Two-sided unpaired *t*-test. **c**, Kaplan–Meier curves with log-rank test show a significant decrease in the survival of *Adh5*^−/−^*Aldh2*^+/KI^*Csb*^−/−^ animals compared to *Adh5*^−/−^*Aldh2*^+/KI^ or *Adh5*^−/−^*Csb*^−/−^ animals (*P* < 0.0001). **d**, Quantification of HSCs (Lin^−^c-Kit^+^Sca-1^+^CD150^+^CD48^−^) in the indicated genotype animals (means ± s.e.m.; *n* = 7 for wild-type, *n* = 6 for *Adh5*^*+*/−^*Aldh2*^*+*/KI^, *n* = 6 for *Adh5*^−/−^*Aldh2*^+/KI^, *n* = 3 for *Adh5*^−/−^*Aldh2*^KI/KI^, *n* = 7 for *Adh5*^−/−^*Csb*^−/−^, *n* = 4 for *Adh5*^*+*/−^*Aldh2*^*+*/KI^*Csb*^−/−^, *n* = 5 for *Adh5*^*+*/−^*Aldh2*^KI/KI^*Csb*^−/−^, *n* = 4 for *Adh5*^−/−^*Aldh2*^+/KI^*Csb*^−/−^). Statistical significance was evaluated with Dunnett’s multiple comparison test. *P* compared to *Adh5*^−/−^*Aldh2*^+/KI^*Csb*^−/−^ mice. BM, bone marrow. **e**, Metagene profile from DPC-seq within or near transcribed regions of genes with TPM ≥ 30 (left) and 0.01 ≤ TPM < 0.1 (right). Data represent the average of three mice. **f**, DPC-seq read counts (reads per gene length per million) are shown in six TPM bins (left) and in seven gene-length bins (right). Means (±s.e.m.) from three mice are shown. Two-sided unpaired *t*-test. Source numerical data are available in the source data.

**Extended Data Fig. 8:**
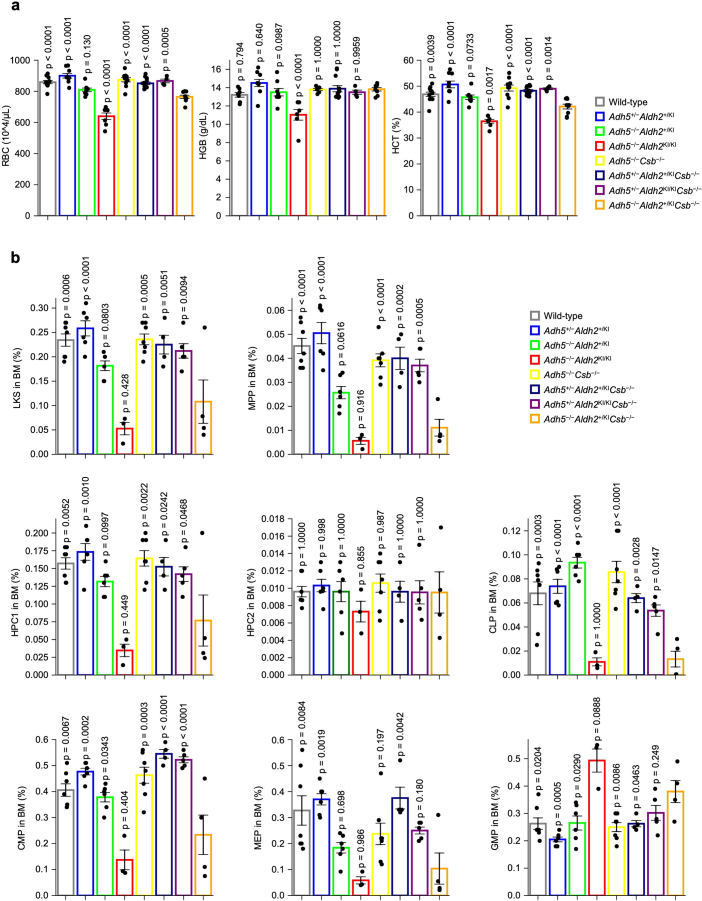
Loss of *Csb* exacerbates abnormal hematopoiesis in aldehyde metabolism-deficient AMeDS mice. (**a**) Red blood cell (RBC), hemoglobin concentration (HGB), hematocrit (HCT), in peripheral blood at 3 to 4 weeks of age were analysed. (mean ± s.e.m., RBC and HCT: n = 11 for wild-type, n = 9 for *Adh5*^*+*/−^*Aldh2*^*+*/KI^, n = 8 for *Adh5*^−/−^*Aldh2*^+/KI^, n = 7 for *Adh5*^−/−^*Aldh2*^KI/KI^, n = 10 for *Adh5*^−/−^*Csb*^−/−^, n = 13 for *Adh5*^*+*/−^*Aldh2*^*+*/KI^*Csb*^−/−^, n = 4 for *Adh5*^*+*/−^*Aldh2*^KI/KI^*Csb*^−/−^, n = 9 for *Adh5*^−/−^*Aldh2*^+/KI^*Csb*^−/−^, HGB: n = 7 for wild-type, n = 9 for *Adh5*^*+*/−^*Aldh2*^*+*/KI^, n = 8 for *Adh5*^−/−^*Aldh2*^+/KI^, n = 6 for *Adh5*^−/−^*Aldh2*^KI/KI^, n = 6 for *Adh5*^−/−^*Csb*^−/−^, n = 12 for *Adh5*^*+*/−^*Aldh2*^*+*/KI^*Csb*^−/−^, n = 4 for *Adh5*^*+*/−^*Aldh2*^KI/KI^*Csb*^−/−^, n = 8 for *Adh5*^−/−^*Aldh2*^+/KI^*Csb*^−/−^). Statistical significance was evaluated with Dunnett’s multiple comparison test. *P* compared to *Adh5*^−/−^*Aldh2*^+/KI^*Csb*^−/−^ mice. (**b**) Quantification of hematopoietic subset: LKS (Lin^−^c-Kit^+^Sca-1^+^), MPP (Lin^−^c-Kit^+^Sca-1^+^CD150^−^CD48^−^), HPC1 (Lin^−^c-Kit^+^Sca-1^+^CD150^−^CD48^+^), HPC2 (Lin^−^c-Kit^+^Sca-1^+^CD150^+^CD48^+^), CLP (Lin^−^c-Kit^low^Sca-1^low^CD127^+^CD135^+^), CMP (Lin^−^c-Kit^+^Sca-1^−^CD34^+^CD16/32^−^), MEP (Lin^−^c-Kit^+^Sca-1^−^CD34^−^CD16/32^−^), and GMP (Lin^−^c-Kit^+^Sca-1^−^CD34^+^CD16/32^+^) in mice at 3 to 4 weeks of age is shown (means ± s.e.m.; n = 7 for wild-type, n = 6 for *Adh5*^+/−^*Aldh2*^*+*/KI^, n = 6 for *Adh5*^−/−^*Aldh2*^+/KI^, n = 3 for *Adh5*^−/−^*Aldh2*^KI/KI^, n = 7 for *Adh5*^−/−^*Csb*^−/−^, n = 4 for *Adh5*^*+*/−^*Aldh2*^*+*/KI^*Csb*^−/−^, n = 5 for *Adh5*^*+*/−^*Aldh2*^KI/KI^*Csb*^−/−^, n = 4 for *Adh5*^−/−^*Aldh2*^+/KI^*Csb*^−/−^). Statistical significance was evaluated with Dunnett’s multiple comparison test. *P* compared to *Adh5*^−/−^*Aldh2*^+/KI^*Csb*^−/−^ mice. Source numerical data are available in source data.

To test this, we quantified DPC levels in lineage-depleted (Lin^−^) bone marrow cells prepared from *Adh5* and *Aldh2* double-deficient mice (Fig. 6a). Bone marrow failure is the most prominent phenotype in these animals, as previously reported^11^. Notably, we detected a statistically significant increase in DPC levels in *Adh5*^−/−^*Aldh2*^KI/KI^ mice compared to wild-type mice (Fig. 6b). To understand the role of TCR in removing DPCs in mice, we generated animals compromised in both aldehyde detoxification and the TCR pathway. We generated triple-deficiency animals with the *Adh5*^−/−^*Aldh2*^+/KI^*Csb*^−/−^ genotype by crossbreeding *Adh5*^+/−^*Csb*^−/−^ mice with *Adh5*^+/−^*Aldh2*^KI/KI^*Csb*^−/−^ mice. This approach was necessary because of devastating phenotypes of the *Adh5*^−/−^*Aldh2*^KI/KI^ double-null mutations^11^; no pups with the *Adh5*^−/−^*Aldh2*^KI/KI^*Csb*^−/−^ genotype were observed from parental animals with the *Adh5*^+/−^*Aldh2*^KI/KI^*Csb*^−/−^ genotype (0 out of 26, *P* = 0.013; *χ*^2^ test). Animals with the *Adh5*^−/−^*Aldh2*^+/KI^*Csb*^−/−^ genotype were born at a significantly lower frequency than expected (12.7%, instead of 25%), whereas animals with the *Adh5*^−/−^*Csb*^−/−^, *Adh5*^−/−^*Aldh2*^+/KI^ and *Adh5*^−/−^*Aldh2*^K/KI^ genotypes were born at the expected Mendelian rate (Extended Data Fig. 7b–d). The *Adh5*^−/−^*Aldh2*^+/KI^*Csb*^−/−^ genotype animals displayed a short life span and growth failure with poor weight gain at 2 weeks and 6–7 months after birth (Fig. 6c and Extended Data Fig. 7e). Moreover, because *Adh5*^−/−^*Aldh2*^+/KI^*Csb*^−/−^ mice exhibited anaemia, similar to *Adh5*^−/−^*Aldh2*^KI/KI^ mice (Extended Data Fig. 8a), we conducted flow cytometric analyses of bone marrow cells from these animals. We found that the number of multipotent self-renewing haematopoietic stem cells (HSCs) defined by Lin^−^c-Kit^+^Sca-1^+^CD150^+^CD48^−^ (CD150^+^ long-term HSCs), immature haematopoietic progenitors, including Lin^−^c-Kit^+^Sca-1^+^CD150^−^CD48^−^ (CD150^−^ multipotent progenitors (MPPs)) and Lin^−^c-Kit^+^Sca-1^+^CD150^−^CD48^+^ (CD48^+^ restricted progenitors (HPC1)) cells were reduced in *Adh5*^−/−^*Aldh2*^+/KI^*Csb*^−/−^ mice (Fig. 6d and Extended Data Fig. 8b). Regarding more differentiated progenitor cells, Lin^−^c-Kit^low^Sca-1^low^CD127^+^CD135^+^ (common lymphoid progenitors (CLPs)), Lin^−^c-Kit^+^Sca-1^−^CD34^+^CD16/32^−^ (CD34^+^ common myeloid progenitors (CMPs)) and Lin^−^c-Kit^+^Sca-1^−^CD34^−^CD16/32^−^ (bipotent megakaryocyte/erythrocyte lineage-restricted progenitors (MEPs)) were also substantially diminished in *Adh5*^−/−^*Aldh2*^+/KI^*Csb*^−/−^ mice (Extended Data Fig. 8b). Overall, TCR deficiency in animals compromised in aldehyde clearance mechanisms exacerbates AMeDS manifestations, including haematopoietic abnormalities, highlighting the importance of the TCR pathway in resolving DNA damage induced by endogenous aldehydes.

### TCR is required for the removal of DPCs in mice

**Extended Data Fig. 9:**
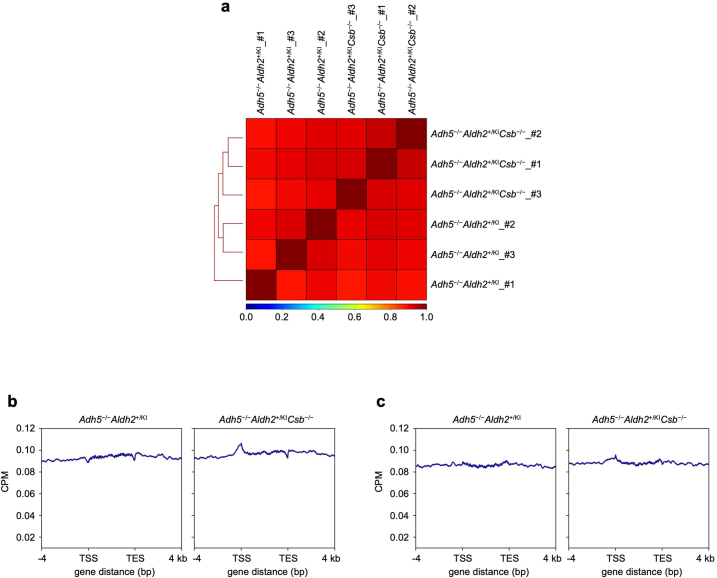
DPC-seq in mice. (**a**) A hierarchically clustered heatmap showing Spearman’s rank-correlation coefficients of DPC-seq signal intensities (CPM) between *Adh5*^–/–^*Aldh2*^+/KI^ and *Adh5*^–/–^*Aldh2*^+/KI^*Csb*^–/–^ mice. (**b** and **c**) Metagene profile from input samples around the transcribed regions of genes with TPM ≥ 30 (b) and 0.01 ≤ TPM < 0.1 (c). TSS: transcription start site; TES: transcription end site. Data represent the average of two mice.

To investigate whether DPCs are repaired by the TCR pathway at the individual animal level, we performed in vivo DPC-seq to measure the distribution of aldehyde-derived endogenous DPCs in Lin^−^ bone marrow cells. Compared to animals with the control *Adh5*^−/−^*Aldh2*^+/KI^ genotype, *Adh5*^−/−^*Aldh2*^+/KI^*Csb*^−/−^ mice displayed a striking increase in their DPC-seq signals, especially within the gene bodies of highly transcribed genes (TPM ≥ 30). In contrast, the signals in inactive genes remained unchanged across the genome. These observations were highly reproducible between animals (Fig. 6e and Extended Data Fig. 9a–c). Remarkably, DPCs were accumulated significantly in *Adh5*^−/−^*Aldh2*^+/KI^*Csb*^−/−^ mice compared to animals with the control genotype, especially in shorter genes, as well as in actively transcribed genes (Fig. 6e,f). In conclusion, endogenously generated DPCs in mice deficient in the aldehyde clearance metabolic processes are removed by the *Csb*-dependent TCR pathway.

## Discussion

In this Article we have identified a previously uncharacterized DNA repair pathway, namely transcription-coupled DNA–protein crosslink repair (TC-DPCR), which is responsible for the prompt and efficient removal of DPCs from actively transcribed regions.

### Development of DPC-seq

We have developed an NGS technique to measure the genome-wide DPC distribution. DPC-seq successfully enabled us to measure DPC repair kinetics in individual genes as well as the metagenome. The profiles appear to reflect the distribution of chromatin-bound histones, revealing the absence of DPC signals in nucleosome-free promoter/TSS regions of active genes. DPC-seq can also measure endogenous DPCs in mouse haematopoietic tissues. The method can be applied to study the effects of aldehyde metabolism in various organs, identify genes susceptible to aldehyde toxicity, and measure the repair kinetics of DPCs induced by reagents other than reactive aldehydes, such as 5′-aza-2-deoxycytidine (5-aza-dC) or TOP1 inhibitors. This is particularly relevant if DPCs generated by these reagents are removed by TCR.

### Mechanistic insights into the resolution of DPCs by TCR

TCR is triggered by RNAPII stalling at transcription roadblocks induced by various genotoxic stresses. However, the majority of previous works have focused on UV damage and its repair mechanisms by TC-NER. Our Article reveals that formaldehyde also activates the CSB-dependent conventional TCR pathway, involving the ubiquitination of RPB1 and UVSSA-dependent recruitment of TFIIH. In this process, proteins crosslinked with DNA are degraded to remnant peptides by the VCP/p97 and proteasome axis, before removal by NER.

Δ*UVSSA* or *POLR2A*-K1268R cells showed milder cellular phenotypes compared to Δ*CSB* cells, raising the possibility that the removal of aldehyde-induced DPCs may involve an alternative repair process distinct from that for UV damage. Although the interaction mechanism between stalled RNAPII and TCR initiation factors after formaldehyde treatment resembles that after UV irradiation, the recruitment of TFIIH to formaldehyde-induced stalled RNAPII is less pronounced. Our data show that histones are crosslinked with DNA (histone-DPCs) following formaldehyde exposure, leading to the obstruction of RNAPII progression. Unlike UV damage, where RNAPII stalls and triggers TC-NER when a photolesion in the DNA template reaches the catalytic centre^46^, aldehyde-induced histone-DPCs never enter the polymerase’s active site due to their large size, and the elongating RNAPII may stall just before encountering the damage. This spatial difference between DNA damage and stalled RNAPII may provide TCR initiation factors with an opportunity to recruit distinct downstream DNA repair machineries in response to aldehyde-induced DNA damage. In fact, our data and the accompanying papers^47,48^ demonstrate that *XPA*-deficient cells are proficient in RRS following formaldehyde treatment. This emphasizes that the removal processes of remnant peptides may require additional factors that recognize large-sized DNA lesions and/or alternative incision machineries.

The major sources of DPCs are presumably conjugations of histones with DNA^39,40^. Although histone proteins are relatively smaller in size (11–22 kDa), they may still need to be degraded into much smaller peptides before being recognized and removed by NER in humans, because the previously reported upper size limit of DPCs acceptable to NER, tested biochemically, is ~10 kDa (refs. ^26,27^). Our data presented here demonstrate that the proteolytic degradation of DPCs through the proteasome, after unfolding by VCP/p97, is an imperative process for the rapid removal of formaldehyde-induced damage from active genes. This suggests that histone-DPCs are removed after degradation to remnant peptides during TC-DPCR. Another potential source of DPCs is RNAPII itself. As prolonged stalling of RNAPII may trigger its degradation in dependence on CSB^30^, we presume that RNAPII crosslinked with DNA should also be ubiquitinated by CSB before being targeted by VCP/p97. However, based on the data presented in the Results, this contribution is negligible. We expect that the majority of the stalled RNAPII is not crosslinked with DNA following formaldehyde treatment, and the RNAPII-p97 interaction can be explained by the existence of other crosslinked proteins, that is, histone-DPCs, located in front of the stalled RNAPII.

Nucleosomes are a strong barrier to RNAPII progression due to steric interference, leading to the stalling of RNAPII and backtracking in vitro. TFIIS promotes transcription resumption by stimulating RNAPII’s intrinsic RNA cleavage activity. Transcription elongation can also be disturbed by DNA lesions, including UV-induced cyclobutane pyrimidine dimers (CPD) and bulky DNA adducts. Although TFIIS supports RNAPII backtracking and transcription restart stalled at CPD in vitro^49,50^, its role at transcription-blocking DNA lesions in vivo remains elusive^51,52^. Our findings reveal that nucleosomes become crosslinked with DNA in cells exposed to formaldehyde, and TFIIS is exclusively recruited to stalled RNAPII at aldehyde-derived DNA damage but not at UV lesions. Furthermore, TFIIS may be particularly important for resuming transcription elongation when RNAPII encounters histone-DPCs, similar to its role in navigating nucleosome barriers^53,54^. However, our data suggest that TFIIS and CSB operate independently in the resumption of arrested RNAPII following formaldehyde treatment. This raises the possibility of alternative explanations, such as the induction of formaldehyde-induced non-DPC DNA lesions, for example, ICLs and base adducts. Further research is needed to address these questions.

### Mouse model and human disorders associated with DPC removal

We have demonstrated that the overload of DNA repair pathways with DPCs causes transcriptional stress, and, in parallel, TCR deficiency aggravates the effects of aldehyde toxicity. In our animal model, lacking both aldehyde clearance metabolic processes (*Adh5*^*−/−*^*Aldh2*^+/KI^) and the TCR pathway (*Csb*^*−/−*^), symptoms of AMeDS are exacerbated by TCR deficiency; concurrently, symptoms of CS are highlighted due to aldehyde clearance deficiency. Our data suggest that TCR is as important as metabolic clearance for protecting against endogenous aldehydes in animals.

Haematopoietic failure is a major phenotype that is common to both FA and AMeDS. It is obvious that endogenous aldehydes underlie the development of both diseases, as the genotype of the rs671 allele, which determines the aldehyde detoxification status of ALDH2, correlates with the severity of their manifestations^11,17^. AMeDS is caused by an increase in endogenous aldehydes, which damages DNA, due to deficiencies in aldehyde-detoxifying enzymes, whereas FA is caused by abnormalities in the FA pathway, which repairs DNA damage caused by aldehydes. As the FA pathway primarily repairs ICL, the main cause of haematopoietic failure in FA and AMeDS is believed to be the accumulation of unrepaired ICL, leading to the inhibition of HSC proliferation. Although moderate AMeDS mice (*Adh5*^*−/−*^*Aldh2*^+/KI^) exhibit haematopoietic failure in adults^11^, the additional loss of TCR exacerbates this manifestation in juvenile animals. The aggravated blood phenotypes may be explained by the unrepaired DPCs in transcribed regions. Note that AMeDS patients display additional manifestations, not observed in FA, such as neurological abnormalities and dwarfism, reminiscent of TCR-deficient CS. This suggests that the accumulation of aldehyde-induced DNA damage repaired by pathways other than FA is relevant to AMeDS disease development. Considering the data presented here, which indicate the involvement of TCR in removing DPCs, it is apparent that the common clinical manifestations observed in AMeDS, CS and RJALS—caused by DPC repair deficiency—can be explained by a shared molecular pathology^3,11^: the overload of DPC repair pathways with endogenous aldehyde-induced DNA damage.

## Methods

The research conducted in this study complies with all relevant ethical regulations. Experiments using genetically modified mice and recombinant DNA were approved by the Animal Care and Use Committee and the Recombinant DNA Experiment Committee of Nagoya University (approval nos. R230006, 166-101 and 169-113).

### Plasmid and siRNA

The full-length human *TFIIS* cDNA (clone ID 6470030, Horizon Discovery) was cloned into pEGFP-C1 (Clontech) containing the N-terminal Strep-V5-tag. The *TFIIS* cDNAs were rendered insensitive to *TFIIS* siRNA by introducing the underlined silent mutations (5′- aacagGaaAgaCgaAacaaat-3′). Cloning and site-directed mutagenesis were performed using KOD-Plus-Neo (TOYOBO). All constructs were verified by Sanger sequencing. Plasmid DNA and siRNA transfections were performed using X-tremeGENE HP (Roche) and Lipofectamine RNAiMAX (Thermo Fisher Scientific), respectively, according to the manufacturer’s instructions. The siRNA target sequences used in this study are listed in Supplementary Table 7.

### Cell lines and culture

HeLa cells (laboratory stock) and 293FT cells (Thermo Fisher Scientific) were maintained in Dulbecco’s modified Eagle medium (DMEM, FUJIFILM) supplemented with 10% fetal bovine serum (FBS, Invitrogen) and antibiotics, unless otherwise noted. Mycoplasma testing was performed routinely. Cell lines stably expressing Strep-V5-GFP-TFIIS were obtained by selecting HeLa cells transfected with pEGFP-C1-Strep-V5-GFP-TFIIS plasmid in medium containing 500 μg ml^−1^ G418 (Nacalai Tesque). HeLa Δ*CSB* cells were transfected with pEGFP-C1-Strep-V5-GFP-CSB plasmid in medium containing 500 μg ml^−1^ G418 (Nacalai Tesque). Unless otherwise indicated, the following doses of chemicals were used: CB5083 (10 μM, Selleck), DRB (100 μM, TCI), epoxomicin (1 μM, FUJIFILM), formaldehyde (600 μM, Nacalai Tesque), MG262 (1 μM, AdipoGen Life Sciences), NMS873 (10 μM, Sigma-Aldrich) and triptolide (3 μM, AdipoGen Life Sciences).

### Genome editing using CRISPR-Cas9 technology

Δ*CSB*, Δ*CSA*, Δ*UVSSA* and *POLR2A*^KR^ cells have been described previously^31^. To edit the *FANCA* gene, HiFi Cas9 nuclease V3 (Integrated DNA Technologies) was mixed with CRISPR RNA (crRNA):trans-activating CRISPR RNA (tracrRNA) complex. The mixture was electroporated into HeLa cells using 4D-Nucleofector (Lonza). Cells were recovered by DMEM with 10% FBS and cultured on a 35-mm dish for 24 h. Single-cell clones were isolated using limiting dilution in 96-well plates. To edit the *XPA* gene, a guide RNA (gRNA) coding sequence was cloned into the PX459 vector^55^. HeLa cells were transfected with the plasmid using Lipofectamine 2000 (Thermo Fisher Scientific). Cells were selected for 48 h in medium containing 2 μg ml^−1^ puromycin (Nacalai Tesque). Single clones were isolated by limiting dilution. Mutations of gene-edited cells were confirmed by Sanger sequencing. The crRNA, gRNA, and primer sequence information is listed in Supplementary Table 7.

### Immunochemical methods

To analyse whole cell lysate, cells were dissolved in Laemmli sodium dodecyl sulfate (SDS) sample buffer (50 mM Tris pH 6.8, 10% glycerol, 100 mM dithiothreitol (DTT), 2% SDS, 0.1% bromophenol blue) and cleared by centrifugation. Immunoprecipitation with anti-RPB1-phospho-Ser2-CTD antibodies was performed as described in ref. ^31^. Briefly, cells were suspended in EBC buffer (50 mM Tris pH 7.5, 150 mM NaCl, 0.5% NP-40, 2 mM MgCl_2_) supplemented with protease inhibitor cocktail (Roche) and phosphatase inhibitor cocktail (Nacalai Tesque). After centrifugation for 5 min at 4 °C, the supernatants were collected (nuclear/cytoplasmic fraction), and the pellets were further incubated in EBC buffer with Benzonase (Merck) for 1 h at 4 °C, followed by centrifugation (chromatin fraction). The extract was then incubated with anti-RPB1-phospho-Ser2-CTD antibodies (ab5095, abcam) and protein A agarose beads (Milipore, #16-157) for 3 h on a rotator at 4 °C. After an extensive wash in lysis buffer, the immunoprecipitates were eluted with 2× Laemmli sample buffer. A Strep-Tactin pulldown assay was carried out as described in ref. ^56^. Briefly, 293FT cells were transfected with plasmid harbouring Strep-Myc-tagged ubiquitin using X-tremeGENE HP (Roche) according to the manufacturer’s instructions. Cells were lysed in denaturing buffer (20 mM Tris, pH 7.5, 50 mM NaCl, 1 mM EDTA, 1 mM DTT, 0.5% NP-40, 0.5% sodium deoxycholate and 0.5% SDS) supplemented with protease and phosphatase inhibitors. After sonication and clearing of the lysates, the supernatant was incubated with Strep-Tactin Sepharose (IBA Lifesciences) for 3 h at 4 °C. After extensive washing with denaturing buffer, the precipitates were eluted with 2× Laemmli sample buffer. Proteins were resolved by SDS–polyacrylamide gel electrophoresis (PAGE). Resolved protein samples were transferred to polyvinylidene fluoride membrane for immunodetection. The antibodies used for immunochemical experiments are listed in Supplementary Table 8.

### MS analysis

Enriched proteins using anti-RPB1-phospho-Ser2-CTD antibodies (ab5095, abcam) or anti-TFIIS antibodies (302–239A, Bethyl Laboratories) were digested after alkylation using the filter-aided sample preparation protocol^57^. Briefly, the immunoprecipitates were dissolved in SDS buffer (0.1 M Tris pH 8.5, 2% SDS, 100 mM DTT) and boiled for 5 min. The proteins were diluted in 8 M urea in 0.1 M Tris pH 8.5, and loaded onto 30-kDa Vivacon 500 centrifugal units (Sartorius). The buffer was replaced by washing with urea buffer, and the proteins were alkylated with 50 mM iodoacetamide for 20 min at 25 °C in the dark. The proteins were then digested with Lys-C (FUJIFILM) and trypsin (Thermo Fisher Scientific) overnight at 37 °C. The resulting peptides were purified using C18 tips (AMR). The peptides from the immunoprecipitates were analysed by liquid chromatography mass spectrometry (LC-MS) using an Orbitrap Fusion mass spectrometer (Thermo Fisher Scientific) coupled to an UltiMate3000 RSLCnano LC system (Dionex Co.) using a nano-HPLC capillary column (150 mm × 75 m i.d., Nikkyo Technos Co.) via a nanoelectrospray ion source. Reversed-phase chromatography was performed with a linear gradient (0 min, 5% B; 75 min, 35% B) of solvent A (2% acetonitrile with 0.1% formic acid) and solvent B (95% acetonitrile with 0.1% formic acid) at an estimated flow rate of 300 nl min^−1^. A precursor ion scan was carried out using a 400–1,600 mass-to-charge ratio (*m*/*z*) before MS/MS analysis. Tandem MS was performed by isolation at 0.8 Th with the quadrupole, higher-energy collisional dissociation (HCD) fragmentation with a normalized collision energy of 30%, and rapid scan MS analysis in the ion trap. Only those precursors with charge state 2–6 were sampled for MS2. The dynamic exclusion duration was set to 15 s with a 10-ppm tolerance. The instrument was run in top speed mode with 3-s cycles. All experiments were performed in the data-dependent acquisition mode to automatically isolate and fragment top ten multiply-charged precursors (+2, +3, +4 and +5) according to their intensities. Former target ions were dynamically excluded for 15 s, and all experiments were acquired using the positive polarity mode. The full scan resolution was set to 70,000, and the mass range was set to *m*/*z* 350–1,400. The full scan ion target value was set to 3 × 10^6^, allowing a maximum fill time of 60 ms. HCD fragment scans were acquired with the optimal setting for parallel acquisition using a 1.6 *m*/*z* isolation window and a normalized collision energy of 27.

### Proteomic data analysis

Raw data were analysed with MaxQuant software (version 1.6.0.1). A UniProt database for the human proteome (Proteome ID UP000005640) was used to search for precursor ions and MS/MS spectra using the Andromeda search engine^58^. Carbamidomethylation of cysteines was searched as a fixed modification, and oxidation of methionines and acetylation of protein N termini as variable modifications. Enzyme specificity was set to trypsin and Lys-C, and a maximum of two missed cleavages were allowed for searching with a precursor mass tolerance of 4.5 ppm and a fragment mass tolerance of 20 ppm. A false discovery rate (FDR) of 0.01 for proteins and peptides and a minimum peptide length of seven amino acids were required. Quantification in MaxQuant was performed using the built-in label-free quantification (LFQ) algorithm. LFQ intensities were analysed with the statistical software package Perseus (version 1.6.0.7). The logarithmized LFQ intensities of the compared conditions were filtered to have two valid values in at least one sample group. Missing values were imputed by creating a normal distribution with a width of 0.3 relative to the standard deviation of the measured values and a 1.8-standard-deviation downshift of the mean. Significant differences in protein abundance were assessed by an unpaired two-sided *t*-test with permutation-based FDR < 0.05. The proteomic data were visualized using R (version 4.0.3) in RStudio (version 2022.02.3 Build 492).

### Measurement of BrU incorporation by flow cytometry

**Extended Data Fig. 10:**
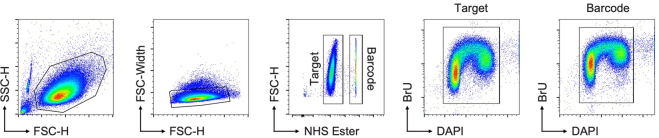
A FACS gating strategy. A flow cytometry gating strategy is described in the Methods section.

For the measurement of RRS after formaldehyde DNA damage, cells were treated with 1,200 μM HCHO for 1 h, followed by 12-h incubation for recovery. Cells were labelled with 1 mM 5-bromo-uridine (BrU) for 1 h, followed by fixing in 4% formaldehyde for 15 min, then permeabilized with PBS containing 0.25% Triton X-100 for 10 min on ice. After washing with PBS containing 0.05% Tween20, the cells were stained with 2 μg ml^−1^ Alexa Fluor 647 NHS Ester (A37573, Thermo Fisher Scientific) for 30 min at room temperature. After washing with PBS containing 0.05% Tween20, stained cells were mixed at a 1:1 ratio with unstained cells. These cells were then stained with Alexa Fluor 488 anti-BrdU antibodies, and the nuclei were stained with 1 μg ml^−1^ 4′,6-diamidino-2-phenylindole (DAPI; D523, DOJINDO). Data were acquired on a CytoFLEX S FACS analyser (Beckman Coulter) by CytExpert (version 2.0) and analysed with FlowJo software (version 10.8.1, BD). The gating strategy is provided in Extended Data Fig. 10.

### RRS assay using 5-ethynyluridine

Details of the assay have been described previously^31^. Briefly, cells were cultured in DMEM supplemented with 10% FBS and plated in plastic 96-well plates. Cells were treated with formaldehyde or irradiated with UV light (254 nm UVC), followed by incubation for RRS. The RRS levels were measured by ethynyluridine (EU) incorporation. Recovered cells as well as untreated cells were incubated for 2 h in medium supplemented with 100 μM 5-ethynyluridine (5-EU, Thermo Fisher Scientific), followed by fluorescent-azide conjugation (Click-chemistry, Thermo Fisher Scientific). The cells were fixed and permeabilized for 20 min in PBS containing 2% formaldehyde and 0.5% Triton X-100. After washing with PBS, the cells were then incubated with coupling buffer with 10 μM Alexa Fluor 488 azide (A10266, Thermo Fisher Scientific), 50 mM Tris-HCl (pH 7.3), 4 mM CuSO_4_, 10 mM sodium ascorbate and 30 ng ml^−1^ DAPI (D523, DOJINDO) for 60 min, followed by washing with PBS containing 0.05% Tween20. Nuclear fluorescent image acquisition and data processing were automated with HCS Studio 2.0 software in the ArrayScan VTI system (Thermo Fisher Scientific).

### Clonogenic survival assays

Cells were seeded in six-well plates and treated with formaldehyde for 2 h or irradiated with UV light. After removing the formaldehyde, cells were allowed to grow for nine days. Cells were fixed with methanol and stained with crystal violet, and colonies with more than 50 cells were counted.

### Animals

C57BL/6JJcl mice were purchased from CLEA Japan. The animals were kept under conditions of 23 ± 1 °C, 50 ± 5% humidity and a 12-h:12-h light:dark cycle. They were fed a standard pellet diet (CE-2, CLEA Japan) and tap water ad libitum. Both male and female mice were used. In Fig. 6b,d–f and Extended Data Figs. 8a,b and 9a–c, mice at three to four weeks of age were analysed. In Fig. 6c, mice at two weeks to one year of age were analysed. In Extended Data Fig. 7a, mice at two weeks to three years of age were analysed. In Extended Data Fig. 7b–d, mice at two weeks of age were analysed. In Extended Data Fig. 7e, mice at two weeks to seven months of age were analysed.

### Genome editing of mouse embryos

*Adh5*^−/−^ and *Aldh2*^+/KI^ mice have been described previously in ref. ^11^. To generate *Csb*-deficient mice, the following reagents were purchased: Cas9 Nuclease V3, tracrRNA and crRNA (Integrated DNA Technologies). Pronuclear-stage mouse embryos were prepared by thawing frozen embryos (CLEA Japan), then culturing them in potassium simplex optimized medium (KSOM, ARK Resource). For electroporation, 100–150 embryos (1 h after thawing) were placed into a chamber with 40 μl of serum-free medium (Opti-MEM, Thermo Fisher Scientific) containing 100 ng μl^−1^ Cas9 Nuclease V3 and 100 ng μl^−1^ *Csb* gRNA. They were then electroporated with a 5-mm gap electrode (CUY505P5, NepaGene) in a NEPA21 Super Electroporator (NepaGene). The pulses for the electroporation had a voltage of 225 V, pulse width of 1 ms for mouse embryos, pulse interval of 50 ms, and the numbers of pulses was 4. The first and second transfer pulses had a voltage of 20 V, pulse width of 50 ms, pulse interval of 50 ms, and the number of pulses was 5. Mouse embryos that developed to the two-cell stage after electroporation were transferred into the oviducts of female surrogates anaesthetized with sevoflurane or isoflurane (Mylan). gRNA sequence information is listed in Supplementary Table 7.

### Mice haematological analysis

Red blood cells (RBCs), haemoglobin concentration and haematocrit were measured using an IDEXX ProCyte Dx system (IDEXX Laboratories).

### Bone marrow cell isolation and FACS analysis

Bone marrow cells were flushed from mice femurs and tibias using a 26-G needle followed by passing through a cell strainer in Ca^2^^+^- and Mg^2^^+^-free Hank’s buffered salt solution (HBSS, Gibco) supplemented with 1% heat-inactivated bovine serum (Gibco). The RBCs were lysed by resuspending the cells in RBC lysis buffer (eBioscience) for 5 min on ice. The cells were filtered through a 70-μm cell strainer to obtain a single-cell suspension. The number of cells was measured with a haemocytometer. Lineage-depleted mouse bone marrow cells were prepared using a Direct Lineage Cell Depletion Kit (Miltenyi Biotec). The antibodies used for FACS analysis were as follows: FITC-conjugated lineage cocktail, CD41, FcεRIα, CD117, Sca-1, CD48, CD150, CD135, CD127, CD16/32 and CD34. Antibody staining was performed at 4 °C for 20 min. Dead cells were excluded by staining with 7-AAD (BioLegend). Data were acquired on a CytoFLEX S FACS analyser (Beckman Coulter) by CytExpert 2.0 and analysed with FlowJo v10.8.1. Antibodies used for FACS analysis are listed in Supplementary Table 8.

### DPC quantification and DPC-seq

Protein-conjugated DNA was isolated using a conventional KCl/SDS precipitation method^32^. The cells were lysed in SDS buffer (2% SDS; 20 mM Tris, pH 7.5) followed by sonication using a Covaris M220 system (5% duty factor, 200 cycles per burst, 16 min at 25 °C in microTUBE). The sonicated samples were incubated with KCl buffer (200 mM KCl, 20 mM Tris pH 7.5) on ice for 5 min. After centrifugation at 20,000*g* for 5 min at 4 °C, the supernatant was collected as soluble DNA. Pellets were washed five times by adding KCl buffer followed by incubation at 55 °C for 5 min. After centrifugation, the pellets were dissolved in KCl buffer with 0.04 mg ml^−1^ Proteinase K and incubated at 55 °C for 3 h. After centrifugation at 20,000*g* for 5 min at 4 °C, the supernatant was purified using a MinElute PCR purification kit (QIAGEN). DNA concentration was measured using Qubit dsDNA HS. The amount of DPCs was calculated as the ratio of precipitated DNA to total DNA (precipitated plus soluble DNA).

In the experiments shown in Extended Data Fig. 4d,e, the cells were synchronized as described in ref. ^59^. Cells were grown in the presence of 2 mM thymidine (Sigma-Aldrich) for 24 h, washed with PBS and grown in fresh medium for 9 h. The cells were then cultured in medium with thymidine for 24 h, washed with PBS, and grown in medium with 0.5 μg ml^−1^ nocodazole (Sigma-Aldrich) for 11 h. After release from the nocodazole block, the cells were treated with formaldehyde for 1 h, washed with PBS, and recovered for 4 h in the presence of thymidine to block S-phase entry. Cell synchronization was confirmed by incorporation of 5-ethynyl-2′-deoxyuridine (EdU, Thermo Fisher Scientific). Briefly, 2 h after release from the nocodazole block, cells were labelled with 5 μM EdU for 5 h in the presence of thymidine, followed by fixing in 4% formaldehyde for 10 min and permeabilization with PBS containing 0.5% Triton X-100 for 5 min. After washing with PBS, the cells were then incubated with coupling buffer with 10 μM Alexa Fluor 488 azide (A10266, Thermo Fisher Scientific), 50 mM Tris-HCl (pH 7.3), 4 mM CuSO_4_ and 10 mM sodium ascorbate. The cells were imaged on a BZ-X800 microscope (Keyence).

For the detection of histone-DPCs, cells were incubated in EBC-2 buffer (50 mM Tris pH 7.5, 150 mM NaCl, 0.5% NP-40, 5 mM EDTA, 0.5 mM EGTA) supplemented with protease inhibitor cocktail (Roche) for 20 min on ice. After centrifugation at 10,000*g* for 5 min at 4 °C, the pellets were dissolved in incubation buffer (50 mM Tris pH 7.5, 150 mM NaCl, 1% Triton X-100, 0.1% sodium deoxycholate, 0.1% SDS, 5 mM EDTA, 0.5 mM EGTA, 0.1% BSA) supplemented with protease inhibitor cocktail (Roche), and sonicated using a Covaris M220 system (5% duty factor, 200 cycles per burst, 16 min at 7 °C in microTUBE). After centrifugation at 20,000*g* for 10 min at 4 °C, the supernatant was incubated with anti-H2B (ab1790, abcam) antibodies and protein A and G magnetic beads (Bio-Rad) for 12–16 h on a rotator at 4 °C. After extensive washing, the beads were incubated with SDS buffer (2% SDS, 20 mM Tris pH 7.5) for 15 min at 30 °C. The eluted supernatant was subjected to DPC isolation.

Purified DNA was subjected to NGS. NGS libraries were generated using NEBNext Ultra II DNA Library Prep Kit for Illumina (NEB) or MGIEasy Universal DNA Library Prep Set (MGI). The prepared libraries were sequenced on a HiSeqX platform (Illumina) with 150-bp paired-end reads, a HiSeq2000 platform (Illumina) with 80-bp single-end reads, a DNBSEQ-G400 platform (MGI Tech) with 150-bp paired-end reads or a DNBSEQ-T7 platform with 150-bp paired-end reads.

The method is available from the Protocol Exchange web site^60^.

### Sequence data analysis

NGS sequence reads were adaptor-trimmed using fastp^61^ (version 0.21.0) and aligned to the GRCh38 (human) or mm10 (mouse) reference genomes using bowtie2 (ref. ^62^) (version 2.3.4.3). Uniquely mapped reads were sorted using samtools^63^ (version 1.15.1), and duplicates were removed using picard MarkDuplicates (version 2.25.0, Broad Institute). The sequence data are listed in Supplementary Table 9.

Mapped reads were visualized using Integrated Genome Viewer^64^ (IGV; version 2.14.1). Read coverage and scores were calculated and visualized using deepTools^65^ (version 3.5.1). The mapped reads on a ribosomal RNA unit were visualized according to ref. ^66^. Spearman’s rank-correlation coefficients between experiments were calculated using deepTools.

To analyse the relationship between transcription level/gene length and DPC repair kinetics, the longest transcript for each gene was extracted from the corresponding reference genomes, and genes with regions overlapping another gene were excluded using bedtools^67^ (version 2.27.1). The gene expression profile of HeLa cells was obtained from the Expression Atlas of EMBL-EBI (https://www.ebi.ac.uk/gxa/experiments/E-MTAB-2706/Results) and the gene expression profile of lineage-depleted cells from mice bone marrow was acquired from RNA-seq data in this study (Supplementary Table 10). For read quantification, mapped reads to reference genomes were counted using HTseq^68^ (version 2.0.2) or bedtools. After excluding mitochondrially encoded genes (in both human and mouse) as well as those on sex chromosomes (in mouse), the read counts were normalized according to their gene length, and the sum was rescaled to 1 × 10^6^ per sample.

### RNA-seq

Total RNA from lineage-depleted mouse bone marrow cells (*n* = 3) was extracted using an RNeasy Kit (QIAGEN). RNA was quantified with a Qubit RNA BR Assay Kit (Life Technology), and integrity was evaluated with Agilent Bioanalyzer 2100 (Agilent Technologies). RNA-seq libraries were constructed with an MGIEasy RNA Directional Library Prep Set (MGI) according to the manufacturer’s instructions. Libraries were sequenced with 150-bp paired-end reads on a DNBSEQ-G400 sequencer (MGI Tech). Low-quality reads and adapter sequences were trimmed using fastp software^61^. The reads were mapped to the GRCm38 reference genome using HISAT2^69^ (version 2.1.0), followed by transcript assembly and quantification using StringTie^70^ (version 1.3.4d).

### Statistics and reproducibility

The statistical tests used are indicated in the figure legends. No statistical method was used to predetermine sample size. The sample sizes chosen are consistent with our previous publications. Data were excluded from analysis only in cases of obvious technical failure. Python (version 3.9.13), R (version 4.0.3), JMP (version 16.1.0) and Excel (version 16.80) were used to generate graphs and perform statistical analyses. Most experiments were replicated. All replication attempts were successful. The numbers of replicate experiments are given in the figure legends or in the figures. Samples were not randomized for this study. This study was not blinded except for colony counting.

### Reporting summary

Further information on research design is available in the Nature Portfolio Reporting Summary linked to this Article.

## Online content

Any methods, additional references, Nature Portfolio reporting summaries, source data, extended data, supplementary information, acknowledgements, peer review information; details of author contributions and competing interests; and statements of data and code availability are available at 10.1038/s41556-024-01401-2.

### Supplementary information

Reporting Summary

Supplementary Tables Supplementary Tables 1–10.

### Source data

Source Data Fig. 2 Unprocessed western blots.

Source Data Fig. 3 Unprocessed western blots.

Source Data Fig. 5 Unprocessed western blots.

Source Data Extended Data Fig. 2 Unprocessed western blots.

Source Data Extended Data Fig. 3 Unprocessed western blots.

Source Data Extended Data Fig. 4 Unprocessed western blots.

Source Data Extended Data Fig. 5 Unprocessed western blots.

Source Data Extended Data Fig. 6 Unprocessed western blots.

Source Data Extended Data Fig. 6 Alternative unprocessed and processed western blots (ED6f-RPB1).

Statistical Source Data Statistical source data

## Data Availability

All data needed to evaluate the conclusions are present in the paper and/or the Supplementary Information. DPC-seq and RNA-seq data in this study have been deposited with links to BioProject accession no. PRJNA1002083 in the NCBI BioProject database. The mass spectrometry proteomics data have been deposited to the ProteomeXchange Consortium via the PRIDE partner repository with the dataset identifier PXD044310 (ref. ^71^). The GRCh38 human genome can be accessed at https://www.ncbi.nlm.nih.gov/datasets/genome/GCF_000001405.40/. The mm10 mouse genome (GRCm38) can be accessed at https://www.ncbi.nlm.nih.gov/datasets/genome/GCF_000001635.26/. The UP000005640 human proteome can be accessed at https://www.uniprot.org/proteomes/UP000005640. The gene expression profile of HeLa cells can be accessed at https://www.ebi.ac.uk/gxa/experiments/E-MTAB-2706/Results. All other data supporting the findings of this study are available from the corresponding author on reasonable request. Additional data related to this paper may be requested from the authors. Source data are provided with this paper.

## References

[1] Lans H, Hoeijmakers JHJ, Vermeulen W, Marteijn JA (2019). The DNA damage response to transcription stress. Nat. Rev. Mol. Cell Biol..

[2] van den Heuvel D (2021). Repair: from mechanism to human disorder. Trends Cell Biol..

[3] Jia N (2021). Dealing with transcription-blocking DNA damage: repair mechanisms, RNA polymerase II processing and human disorders. DNA Repair.

[4] Zhang L (2010). Formaldehyde and leukemia: epidemiology, potential mechanisms and implications for risk assessment. Environ. Mol. Mutagen..

[5] Heck HD (1985). Formaldehyde (CH_2_O) concentrations in the blood of humans and Fischer-344 rats exposed to CH_2_O under controlled conditions. Am. Ind. Hyg. Assoc. J..

[6] Luo WH, Li H, Zhang Y, Ang CYW (2001). Determination of formaldehyde in blood plasma by high-performance liquid chromatography with fluorescence detection. J. Chromatogr. B.

[7] Reingruber H, Pontel LB (2018). Formaldehyde metabolism and its impact on human health. Curr. Opin. Toxicol..

[8] Barnett SD, Buxton ILO (2017). The role of *S*-nitrosoglutathione reductase (GSNOR) in human disease and therapy. Crit. Rev. Biochem. Mol. Biol..

[9] Wang RS, Nakajima T, Kawamoto T, Honma T (2002). Effects of aldehyde dehydrogenase-2 genetic polymorphisms on metabolism of structurally different aldehydes in human liver. Drug Metab. Dispos..

[10] Dingler FA (2020). Two aldehyde clearance systems are essential to prevent lethal formaldehyde accumulation in mice and humans. Mol. Cell.

[11] Oka Y (2020). Digenic mutations in ALDH2 and ADH5 impair formaldehyde clearance and cause a multisystem disorder, AMeD syndrome. Sci. Adv..

[12] Huang H, Hopkins PB (1993). DNA interstrand cross-linking by formaldehyde - nucleotide-sequence preference and covalent structure of the predominant cross-link formed in synthetic oligonucleotides. J. Am. Chem. Soc..

[13] Voulgaridou GP, Anestopoulos I, Franco R, Panayiotidis MI, Pappa A (2011). DNA damage induced by endogenous aldehydes: current state of knowledge. Mutat. Res..

[14] Che R, Zhang J, Nepal M, Han B, Fei P (2018). Multifaceted Fanconi anemia signaling. Trends Genet..

[15] Ceccaldi R, Sarangi P, D’Andrea AD (2016). The Fanconi anaemia pathway: new players and new functions. Nat. Rev. Mol. Cell Biol..

[16] Garaycoechea JI (2012). Genotoxic consequences of endogenous aldehydes on mouse haematopoietic stem cell function. Nature.

[17] Hira A (2013). Variant ALDH2 is associated with accelerated progression of bone marrow failure in Japanese Fanconi anemia patients. Blood.

[18] Pontel LB (2015). Endogenous formaldehyde is a hematopoietic stem cell genotoxin and metabolic carcinogen. Mol. Cell.

[19] Lessel D (2014). Mutations in SPRTN cause early onset hepatocellular carcinoma, genomic instability and progeroid features. Nat. Genet..

[20] Stingele J, Schwarz MS, Bloemeke N, Wolf PG, Jentsch S (2014). A DNA-dependent protease involved in DNA-protein crosslink repair. Cell.

[21] Stingele J (2016). Mechanism and regulation of DNA-protein crosslink repair by the DNA-dependent metalloprotease SPRTN. Mol. Cell.

[22] Vaz B (2016). Metalloprotease SPRTN/DVC1 orchestrates replication-coupled DNA-protein crosslink repair. Mol. Cell.

[23] Larsen NB (2019). Replication-coupled DNA-protein crosslink repair by SPRTN and the proteasome in *Xenopus* egg extracts. Mol. Cell.

[24] Ortega-Atienza S, Green SE, Zhitkovich A (2015). Proteasome activity is important for replication recovery, CHK1 phosphorylation and prevention of G2 arrest after low-dose formaldehyde. Toxicol. Appl. Pharmacol..

[25] Quievryn G, Zhitkovich A (2000). Loss of DNA-protein crosslinks from formaldehyde-exposed cells occurs through spontaneous hydrolysis and an active repair process linked to proteosome function. Carcinogenesis.

[26] Nakano T (2009). Homologous recombination but not nucleotide excision repair plays a pivotal role in tolerance of DNA-protein cross-links in mammalian cells. J. Biol. Chem..

[27] Reardon JT, Sancar A (2006). Repair of DNA-polypeptide crosslinks by human excision nuclease. Proc. Natl Acad. Sci. USA.

[28] Laugel V (2013). Cockayne syndrome: the expanding clinical and mutational spectrum. Mech. Ageing Dev..

[29] Karikkineth AC, Scheibye-Knudsen M, Fivenson E, Croteau DL, Bohr VA (2017). Cockayne syndrome: clinical features, model systems and pathways. Ageing Res. Rev..

[30] Nakazawa Y (2012). Mutations in UVSSA cause UV-sensitive syndrome and impair RNA polymerase IIo processing in transcription-coupled nucleotide-excision repair. Nat. Genet..

[31] Nakazawa Y (2020). Ubiquitination of DNA damage-stalled RNAPII promotes transcription-coupled repair. Cell.

[32] Zhitkovich A, Costa M (1992). A simple, sensitive assay to detect DNA-protein crosslinks in intact cells and in vivo. Carcinogenesis.

[33] Russell J, Zomerdijk JC (2005). RNA-polymerase-I-directed rDNA transcription, life and works. Trends Biochem. Sci..

[34] Olivieri M (2020). A genetic map of the response to DNA damage in human cells. Cell.

[35] Mulderrig L (2021). Aldehyde-driven transcriptional stress triggers an anorexic DNA damage response. Nature.

[36] van der Weegen Y (2020). The cooperative action of CSB, CSA and UVSSA target TFIIH to DNA damage-stalled RNA polymerase II. Nat. Commun..

[37] van den Boom J, Meyer H (2018). VCP/p97-mediated unfolding as a principle in protein homeostasis and signaling. Mol. Cell.

[38] Kornberg RD (1974). Chromatin structure: a repeating unit of histones and DNA. Science.

[39] O’Connor PM, Fox BW (1989). Isolation and characterization of proteins cross-linked to DNA by the antitumor agent methylene dimethanesulfonate and its hydrolytic product formaldehyde. J. Biol. Chem..

[40] Pachva MC, Kisselev AF, Matkarimov BT, Saparbaev M, Groisman R (2020). DNA-histone cross-links: formation and repair. Front. Cell Dev. Biol..

[41] Ozsolak F, Song JS, Liu XS, Fisher DE (2007). High-throughput mapping of the chromatin structure of human promoters. Nat. Biotechnol..

[42] Schones DE (2008). Dynamic regulation of nucleosome positioning in the human genome. Cell.

[43] Noe Gonzalez M, Blears D, Svejstrup JQ (2021). Causes and consequences of RNA polymerase II stalling during transcript elongation. Nat. Rev. Mol. Cell Biol..

[44] Sheridan RM, Fong N, D’Alessandro A, Bentley DL (2019). Widespread backtracking by RNA Pol II is a major effector of gene activation, 5′ pause release, termination and transcription elongation rate. Mol. Cell.

[45] Zatreanu D (2019). Elongation Factor TFIIS prevents transcription stress and R-loop accumulation to maintain genome stability. Mol. Cell.

[46] Brueckner F, Hennecke U, Carell T, Cramer P (2007). CPD damage recognition by transcribing RNA polymerase II. Science.

[47] Carnie, C. J. et al. Transcription-coupled repair of DNA–protein crosslinks depends on CSA and CSB. *Nat. Cell Biol.*10.1038/s41556-024-01391-1 (2024). 10.1038/s41556-024-01391-1 PMC11098753 38600235

[48] van Sluis, M. et al. Transcription-coupled DNA–protein crosslink repair by CSB and CRL4^CSA^-mediated degradation. *Nat. Cell Biol.*10.1038/s41556-024-01394-y (2024). 10.1038/s41556-024-01394-y PMC11098752 38600236

[49] Donahue BA, Yin S, Taylor JS, Reines D, Hanawalt PC (1994). Transcript cleavage by RNA polymerase II arrested by a cyclobutane pyrimidine dimer in the DNA template. Proc. Natl Acad. Sci. USA.

[50] Tornaletti S, Reines D, Hanawalt PC (1999). Structural characterization of RNA polymerase II complexes arrested by a cyclobutane pyrimidine dimer in the transcribed strand of template DNA. J. Biol. Chem..

[51] Fousteri M, Mullenders LH (2008). Transcription-coupled nucleotide excision repair in mammalian cells: molecular mechanisms and biological effects. Cell Res..

[52] Mackinnon-Roy C, Stubbert LJ, McKay BC (2011). RNA interference against transcription elongation factor SII does not support its role in transcription-coupled nucleotide excision repair. Mutat. Res..

[53] Kireeva ML (2005). Nature of the nucleosomal barrier to RNA polymerase II. Mol. Cell.

[54] Guermah M, Palhan VB, Tackett AJ, Chait BT, Roeder RG (2006). Synergistic functions of SII and p300 in productive activator-dependent transcription of chromatin templates. Cell.

[55] Ran FA (2013). Genome engineering using the CRISPR-Cas9 system. Nat. Protoc..

[56] Oka Y (2014). UBL5 is essential for pre-mRNA splicing and sister chromatid cohesion in human cells. EMBO Rep..

[57] Wisniewski JR, Zougman A, Nagaraj N, Mann M (2009). Universal sample preparation method for proteome analysis. Nat. Methods.

[58] Cox J (2011). Andromeda: a peptide search engine integrated into the MaxQuant environment. J. Proteome Res..

[59] Oka Y, Bekker-Jensen S, Mailand N (2015). Ubiquitin-like protein UBL5 promotes the functional integrity of the Fanconi anemia pathway. EMBO J..

[60] Oka, Y., Nakazawa, Y., Shimada, M. & Ogi, T. DPC-seq: genome-wide mapping of DNA-protein crosslinks (DPC) induced by endogenous aldehydes. *Protocol Exchange*10.21203/rs.3.pex-2574/v1 (2024).

[61] Chen S, Zhou Y, Chen Y, Gu J (2018). fastp: an ultra-fast all-in-one FASTQ preprocessor. Bioinformatics.

[62] Langmead B, Salzberg SL (2012). Fast gapped-read alignment with Bowtie 2. Nat. Methods.

[63] Li H (2009). The Sequence Alignment/Map format and SAMtools. Bioinformatics.

[64] Thorvaldsdottir H, Robinson JT, Mesirov JP (2013). Integrative Genomics Viewer (IGV): high-performance genomics data visualization and exploration. Brief. Bioinform..

[65] Ramirez F (2016). deepTools2: a next generation web server for deep-sequencing data analysis. Nucleic Acids Res..

[66] George SS, Pimkin M, Paralkar VR (2023). Construction and validation of customized genomes for human and mouse ribosomal DNA mapping. J. Biol. Chem..

[67] Quinlan AR, Hall IM (2010). BEDTools: a flexible suite of utilities for comparing genomic features. Bioinformatics.

[68] Putri GH, Anders S, Pyl PT, Pimanda JE, Zanini F (2022). Analysing high-throughput sequencing data in Python with HTSeq 2.0. Bioinformatics.

[69] Kim D, Paggi JM, Park C, Bennett C, Salzberg SL (2019). Graph-based genome alignment and genotyping with HISAT2 and HISAT-genotype. Nat. Biotechnol..

[70] Pertea M (2015). StringTie enables improved reconstruction of a transcriptome from RNA-seq reads. Nat. Biotechnol..

[71] Oka, Y. & Ogi, T. Interactome analysis of elongating RNAPII after induction of DNA damage by IP-MS/MS. *ProteomeXchange* https://proteomecentral.proteomexchange.org/cgi/GetDataset?ID=PXD044310 (2024).

