# Supplementary material for: Endogenous aldehyde-induced DNA–protein crosslinks are resolved by transcription-coupled repair
Source: Nat Cell Biol. 2024 Apr 10;26(5):784–96. doi: 10.1038/s41556-024-01401-2 (PMC11098742; doi:10.1038/s41556-024-01401-2)
Supplement: Supplementary file 1 — Reporting Summary [file 41556_2024_1401_MOESM1_ESM.pdf]

nature portfolio

Corresponding author(s): Tomoo Ogi

Last updated by author(s): Feb 13, 2024

Reporting Summary

Nature Portfolio wishes to improve the reproducibility of the work that we publish. This form provides structure for consistency and transparency in reporting. For further information on Nature Portfolio policies, see our Editorial Policies and the Editorial Policy Checklist.

Statistics

For all statistical analyses, confirm that the following items are present in the figure legend, table legend, main text, or Methods section.

n/a	Confirmed	
☐	☒	The exact sample size (*n*) for each experimental group/condition, given as a discrete number and unit of measurement
☐	☒	A statement on whether measurements were taken from distinct samples or whether the same sample was measured repeatedly
☐	☒	The statistical test(s) used AND whether they are one- or two-sided<br>*Only common tests should be described solely by name; describe more complex techniques in the Methods section.*
☒	☐	A description of all covariates tested
☐	☒	A description of any assumptions or corrections, such as tests of normality and adjustment for multiple comparisons
☐	☒	A full description of the statistical parameters including central tendency (e.g. means) or other basic estimates (e.g. regression coefficient) AND variation (e.g. standard deviation) or associated estimates of uncertainty (e.g. confidence intervals)
☐	☒	For null hypothesis testing, the test statistic (e.g. *F*, *t*, *r*) with confidence intervals, effect sizes, degrees of freedom and *P* value noted<br>*Give P values as exact values whenever suitable.*
☒	☐	For Bayesian analysis, information on the choice of priors and Markov chain Monte Carlo settings
☒	☐	For hierarchical and complex designs, identification of the appropriate level for tests and full reporting of outcomes
☒	☐	Estimates of effect sizes (e.g. Cohen's *d*, Pearson's *r*), indicating how they were calculated

Our web collection on statistics for biologists contains articles on many of the points above.

Software and code

Policy information about availability of computer code

Data collection	FACS analysis: CytExpert 2.0, High-content analysis: HCS Studio 2.0
Data analysis	FACS data: FlowJo (version 10.8.1). Western blot data: ImageJ (version 1.53a). Mass spectrometry data: MaxQuant software (version 1.6.0.1), Perseus (version 1.6.0.7), R (version 4.0.3). Sequencing data: fastp (version 0.21.0), bowtie2 (version 2.3.4.3), samtools (version 1.15.1), picard MarkDuplicates (version 2.25.0), Integrated Genome Viewer (version 2.14.1), deepTools (version 3.5.1), bedtools (version 2.27.1), HTseq (version 2.0.2), Python (version 3.9.13), HISAT2 (version 2.1.0), StringTie (version 1.3.4), RStudio (version 2022.02.3 Build 492).

For manuscripts utilizing custom algorithms or software that are central to the research but not yet described in published literature, software must be made available to editors and reviewers. We strongly encourage code deposition in a community repository (e.g. GitHub). See the Nature Portfolio guidelines for submitting code & software for further information.

Data

Policy information about availability of data

All manuscripts must include a data availability statement. This statement should provide the following information, where applicable:

- Accession codes, unique identifiers, or web links for publicly available datasets
- A description of any restrictions on data availability
- For clinical datasets or third party data, please ensure that the statement adheres to our policy

DPC-seq and RNA-seq data in this study have been deposited with links to BioProject accession number PRJNA1002083 in the NCBI BioProject database. The mass

spectrometry proteomics data have been deposited to the ProteomeXchange Consortium via the PRIDE partner repository with the dataset identifier PXD044310. The GRCh38 human genome can be accessed at https://www.ncbi.nlm.nih.gov/datasets/genome/GCF_000001405.40/. The mm10 mouse genome (GRCm38) can be accessed at https://www.ncbi.nlm.nih.gov/datasets/genome/GCF_000001635.26/. The UP000005640 human proteome can be accessed at https://www.uniprot.org/proteomes/UP000005640. The gene expression profile of HeLa cells can be accessed at https://www.ebi.ac.uk/gxa/experiments/E-MTAB-2706/Results. All other data supporting the finding of this study are available from the corresponding author on reasonable request. Additional data related to this paper may be requested from the authors.

Research involving human participants, their data, or biological material

Policy information about studies with human participants or human data. See also policy information about sex, gender (identity/presentation), and sexual orientation and race, ethnicity and racism.

Reporting on sex and gender	n/a
Reporting on race, ethnicity, or other socially relevant groupings	n/a
Population characteristics	n/a
Recruitment	n/a
Ethics oversight	n/a

Note that full information on the approval of the study protocol must also be provided in the manuscript.

Field-specific reporting

Please select the one below that is the best fit for your research. If you are not sure, read the appropriate sections before making your selection.

☒ Life sciences ☐ Behavioural & social sciences ☐ Ecological, evolutionary & environmental sciences

For a reference copy of the document with all sections, see nature.com/documents/nr-reporting-summary-flat.pdf

Life sciences study design

All studies must disclose on these points even when the disclosure is negative.

Sample size	No statistical method was used to predetermine sample size. The sample sizes chosen are consistent with previous publications (animal experiments: Oka et al., 2020, cellular experiments: Oka et al., 2020 and Nakazawa et al., 2020).
Data exclusions	Data were excluded from analysis only in cases of obvious technical failure.
Replication	Most experiments were replicated. All replication attempts were successful. The number of replicate experiments is given in the figure legends or in the figures.
Randomization	Samples were not randomized for this study because randomization does not influence the experimental outcomes.
Blinding	This study was not blinded except for colony counting because blinding does not influence the experimental outcomes.

Reporting for specific materials, systems and methods

We require information from authors about some types of materials, experimental systems and methods used in many studies. Here, indicate whether each material, system or method listed is relevant to your study. If you are not sure if a list item applies to your research, read the appropriate section before selecting a response.

Materials & experimental systems

n/a	Involved in the study	
☐	☒	Antibodies
☐	☒	Eukaryotic cell lines
☒	☐	Palaeontology and archaeology
☐	☒	Animals and other organisms
☒	☐	Clinical data
☒	☐	Dual use research of concern
☒	☐	Plants

Methods

n/a	Involved in the study	
☒	☐	ChIP-seq
☐	☒	Flow cytometry
☒	☐	MRI-based neuroimaging

Antibodies

Antibodies used

ACTB, Santa Cruz Biotechnology, sc-47778, 1 to 4000
Cleaved PARP (Asp214), Cell Signaling Technology, #5625, 1 to 1000
CSA, Abcam, ab137033, 1 to 500
CSB, Santa Cruz Biotechnology, sc-398022, 1 to 250
FANCA, Bethyl laboratories, A301-980A, 1 to 1000
GFP, Santa Cruz Biotechnology, sc-9996, 1 to 500
H2B, Abcam, ab1790, 1 to 167
KU70, Cell Signaling Technology, #4588, 1 to 1000
KU80, Cell Signaling Technology, #2180, 1 to 1000
Myc, Santa Cruz Biotechnology, sc-40, 1 to 1000
p62, Santa Cruz Biotechnology, sc-48431, 1 to 250
p89, Santa Cruz Biotechnology, sc-271500, 1 to 250
p97, Santa Cruz Biotechnology, sc-57492, 1 to 250
RPB1 phospho-Ser2, Abcam, ab5095, 1 to 500
RPB1 phospho-Ser2, Merck, 04-1571-I, 1 to 1000
SMC3, Bethyl laboratories, A300-060A, 1 to 4000
TFIIS, Bethyl laboratories, A302-239A, 1 to 333
TFIIS, Santa Cruz Biotechnology, sc-393520, 1 to 250
UVSSA, Abnova, H00057654-B01P, 1 to 500
Alexa Fluor 488 BrdU, BioLegend, #364106, 1 to 20
FITC lineage cocktail, BioLegend, #133302, 1 to 5
FITC CD41, BioLegend, #133903, 1 to 200
FITC FcεRIα, BioLegend, #134305, 1 to 200
APC CD117, BioLegend, #105811, 1 to 20
PE Sca-1, BioLegend, #108107, 1 to 40
Brilliant Violet 421 CD48, BioLegend, #103428, 1 to 80
APC/Fire 750 CD150, BioLegend, #115940, 1 to 80
Brilliant Violet 421 CD135, BioLegend, #135313, 1 to 40
PE/Cy7 CD127, BioLegend, #135014, 1 to 20
Brilliant Violet 421 CD16/32, BioLegend, #101332, 1 to 80
APC/Fire 750 CD34, BioLegend, #128614, 1 to 20

Validation

The following antibodies were validated in siRNA-treated or knockout human cells: CSA (Abcam, ab137033), CSB (Santa Cruz Biotechnology, sc-398022), FANCA (Bethyl laboratories, A301-980A), TFIIS (Bethyl laboratories, A302-239A), TFIIS (Santa Cruz Biotechnology, sc-393520), UVSSA (Abnova, H00057654-B01P)

The following antibodies were validated in immunoprecipitation-based experiments using human cells: p62 (Santa Cruz Biotechnology, sc-48431), p89 (Santa Cruz Biotechnology, sc-271500), p97 (Santa Cruz Biotechnology, sc-57492), RPB1 phospho-Ser2 (Abcam, ab5095), RPB1 phospho-Ser2 (Merck, 04-1571-I)

The following antibodies were validated in exogenous expression-based experiments: Myc (Santa Cruz Biotechnology, sc-40)

ACTB, Santa Cruz Biotechnology, sc-47778
https://www.scbt.com/ja/p/beta-actin-antibody-c4
Cleaved PARP (Asp214), Cell Signaling Technology, #5625
https://www.cellsignal.jp/products/primary-antibodies/cleaved-parp-asp214-d64e10-xp-rabbit-mab/5625
GFP, Santa Cruz Biotechnology, sc-9996
https://www.scbt.com/p/gfp-antibody-b-2
H2B, Abcam, ab1790
https://www.abcam.co.jp/products/primary-antibodies/histone-h2b-antibody-chip-grade-ab1790.html
KU70, Cell Signaling Technology, #4588
https://www.cellsignal.jp/products/primary-antibodies/ku70-d10a7-rabbit-mab/4588
KU80, Cell Signaling Technology, #2180
https://www.cellsignal.jp/products/primary-antibodies/ku80-c48e7-rabbit-mab/2180
SMC3, Bethyl laboratories, A300-060A
https://www.thermofisher.com/antibody/product/SMC3-Antibody-Polyclonal/A300-060A
Alexa Fluor 488 BrdU, BioLegend, #364106
https://www.biolegend.com/ja-jp/products/alexa-fluor-488-anti-brdu-antibody-10621?GroupID=BLG8966
FITC lineage cocktail, BioLegend, #133302
https://www.biolegend.com/ja-jp/products/fitc-anti-mouse-lineage-cocktail-with-isotype-ctrl-5803
FITC CD41, BioLegend, #133903
https://www.biolegend.com/ja-jp/products/fitc-anti-mouse-cd41-antibody-5896
FITC FcεRIα, BioLegend, #134305
https://www.biolegend.com/ja-jp/products/fitc-anti-mouse-fcepsilonrialpha-antibody-5949
APC CD117, BioLegend, #105811
https://www.biolegend.com/ja-jp/products/apc-anti-mouse-cd117-c-kit-antibody-72
PE Sca-1, BioLegend, #108107
https://www.biolegend.com/ja-jp/products/pe-anti-mouse-ly-6a-e-sca-1-antibody-228
Brilliant Violet 421 CD48, BioLegend, #103428
https://www.biolegend.com/ja-jp/products/brilliant-violet-421-anti-mouse-cd48-antibody-7327
APC/Fire 750 CD150, BioLegend, #115940
https://www.biolegend.com/ja-jp/products/apc-fire-750-anti-mouse-cd150-slam-antibody-13440

Brilliant Violet 421 CD135, BioLegend, #135313
https://www.biolegend.com/ja-jp/products/brilliant-violet-421-anti-mouse-cd135-antibody-8728
PE/Cy7 CD127, BioLegend, #135014
https://www.biolegend.com/ja-jp/products/pe-cyanine7-anti-mouse-cd127-il-7ralpha-antibody-6192
Brilliant Violet 421 CD16/32, BioLegend, #101332
https://www.biolegend.com/ja-jp/products/brilliant-violet-421-anti-mouse-cd16-32-antibody-8598
APC/Fire 750 CD34, BioLegend, #128614
https://www.biolegend.com/ja-jp/products/apc-fire-750-anti-mouse-cd34-antibody-14762

Eukaryotic cell lines

Policy information about cell lines and Sex and Gender in Research

Cell line source(s)	HeLa-WT (laboratory stock), HeLa CSB-KO, HeLa CSA-KO, HeLa UVSSA-KO, HeLa POLR2A-K1268R, 293FT (Thermo Fisher Scientific) (Nakazawa et al., 2020)<br>HeLa FANCA-KO, HeLa CSB-FANCA-DKO, HeLa XPA-KO, HeLa CSB-KO +CSB-WT, HeLa + TFIIS-WT, HeLa + TFIIS-D282A-E283A (This study)
Authentication	All knockout cells were validated by DNA sequencing and/or western blotting. All cell lines stably expressing exogenous genes were validated by fluorescent microscopy and western blotting.
Mycoplasma contamination	Cell lines were negative for mycoplasma.
Commonly misidentified lines (See ICLAC register)	No commonly misidentified cell lines were used.

Animals and other research organisms

Policy information about studies involving animals; ARRIVE guidelines recommended for reporting animal research, and Sex and Gender in Research

Laboratory animals	Adh5 knockout and Aldh2 knockin C57BL/6JJcl mice were generated using a CRISPR-Cas9 gene-editing approach (Oka et al., 2020). Csb knockout C57BL/6JJcl mice were generated using a CRISPR-Cas9 gene-editing approach (this study). In figures 6b, 6d-f, extended data figures 8a-b, 9a-c, mice at 3 to 4 weeks of age were analysed. In figure 6c, mice at 2 weeks to 1 year of age were analyzed. In extended data figure 7a, mice at 2 weeks to 3 years of age were analyzed. In extended data figures 7b-d, mice at 2 weeks of age were analysed. In extended data figure 7e, mice at 2 weeks to 7 months of age were analysed.
Wild animals	No wild animals were included.
Reporting on sex	Both male and female mice were used in this study. There was no obvious sex-based difference, but sex-disaggregated data have not been specifically analysed.
Field-collected samples	No field-collected samples were used in this study.
Ethics oversight	All the animal studies were conducted in compliance with the ARRIVE guidelines. The experiments using genetically modified mice were approved by the Animal Care and Use Committee and the recombinant DNA experiment committee of Nagoya University.

Note that full information on the approval of the study protocol must also be provided in the manuscript.

Flow Cytometry

Plots

Confirm that:

☒ The axis labels state the marker and fluorochrome used (e.g. CD4-FITC).

☒ The axis scales are clearly visible. Include numbers along axes only for bottom left plot of group (a 'group' is an analysis of identical markers).

☒ All plots are contour plots with outliers or pseudocolor plots.

☒ A numerical value for number of cells or percentage (with statistics) is provided.

Methodology

Sample preparation	HeLa cells were labeled with 1 mM 5-Bromouridine (BrU) for 1 h followed by fixed in 4% formaldehyde for 15 min, permeabilised with PBS containing 0.25% Triton X-100 for 10 min on ice. After washing with PBS containing 0.05% Tween20, cells were stained with 2 μg/ml Alexa Fluor 647 NHS Ester (A37573, Thermo Fisher Scientific) for 30 min at room temperature. After washing with PBS containing 0.05% Tween20, stained cells were mixed at a 1:1 ratio with unstained cells. Then, these cells were stained with Alexa Fluor 488 anti-BrdU antibodies, and nuclei were stained with 1 μg/ml DAPI (D523, DOJINDO). Data were acquired on a CytoFLEX S FACS analyser (Beckman Coulter) by CytExpert (version 2.0) and analysed with FlowJo (version 10.8.1, BD). Mouse bone marrow cells were flushed from femurs and tibias using a 26G needle followed by passing through a cell strainer in Ca2+- and Mg2+-free Hank's buffered salt solution (HBSS; Gibco) supplemented with 1%

heat-inactivated bovine serum (Gibco). RBCs were lysed by resuspending the cells in RBC lysis buffer (eBioscience) for 5 min on ice. Cells were filtered through a 70 μm cell strainer to obtain a single cell suspension. Number of cells was measured with a hemocytometer. Antibodies used for FACS analysis were as follows: FITC-conjugated lineage cocktail, CD41, FcεRIα, CD117, Sca-1, CD48, CD150, CD135, CD127, CD16/32, CD34. Antibody staining was performed at 4 °C for 20 min. Dead cells were excluded by staining with 7-AAD (BioLegend). Data were acquired on a CytoFLEX S FACS analyser (Beckman Coulter) by CytExpert 2.0 and analysed with FlowJo v10.8.1.

Instrument

CytoFLEX S

Software

CytExpert 2.0, FlowJo 10.8.1

Cell population abundance

Post-sort fractions were not analysed.

Gating strategy

In the BrU-incorporation experiments, a population of single cells were gated based on cells size (FSC height versus FSC area). Anti-BrdU antibodies conjugated with Alexa Fluor 488 (BioLegend, #364106) were used to stain BrU positive cells. The gating strategy is provided in Extended Data Fig. 10. Targeted population of mouse bone marrow cells were gated according to the way in our previous paper (Oka et al., 2020).

☒ Tick this box to confirm that a figure exemplifying the gating strategy is provided in the Supplementary Information.
