# Supplementary figures and images for: Endogenous aldehyde-induced DNA–protein crosslinks are resolved by transcription-coupled repair
Source: Nat Cell Biol. 2024 Apr 10;26(5):784–96. doi: 10.1038/s41556-024-01401-2 (PMC11098742; doi:10.1038/s41556-024-01401-2)

**b**

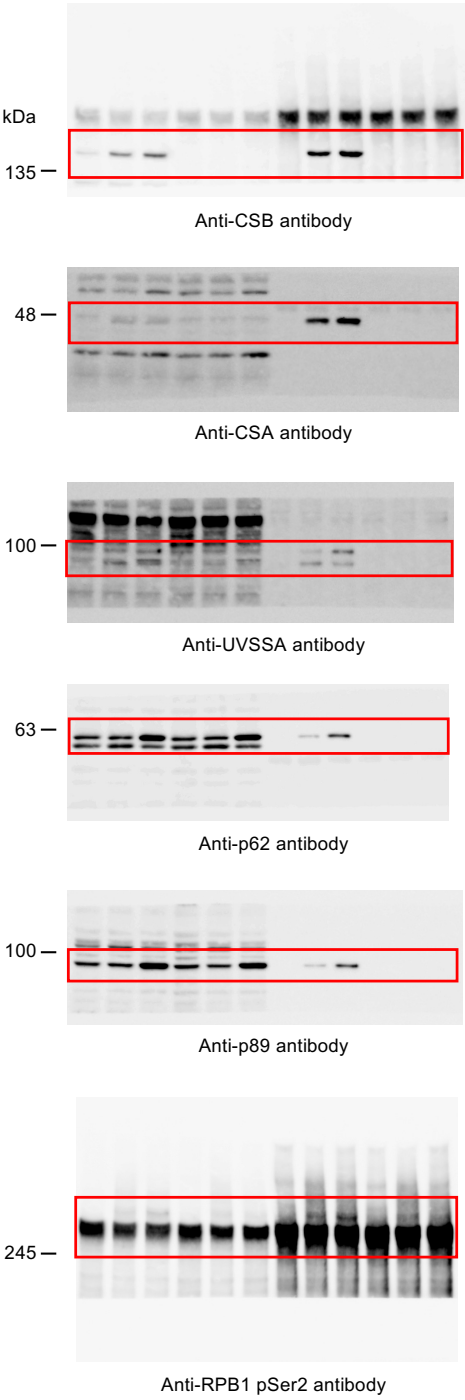

Supplement: Supplementary file 3 — Unprocessed western blots. [file 41556_2024_1401_MOESM3_ESM.pdf]

**b**

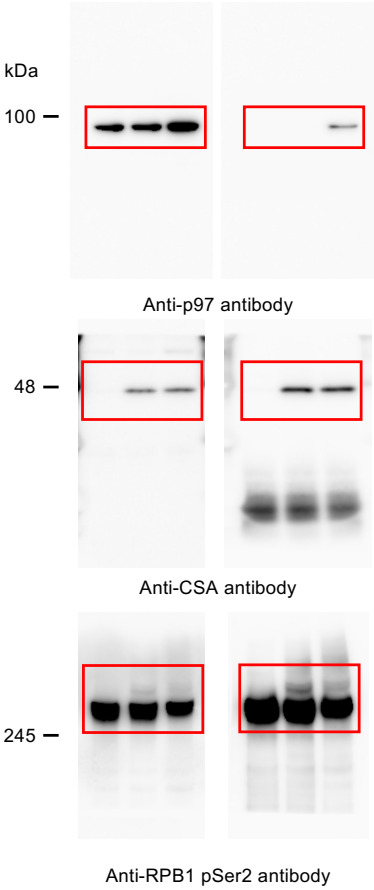

Supplement: Supplementary file 4 — Unprocessed western blots. [file 41556_2024_1401_MOESM4_ESM.pdf]

Fig. 5 uncropped images

Oka et al.

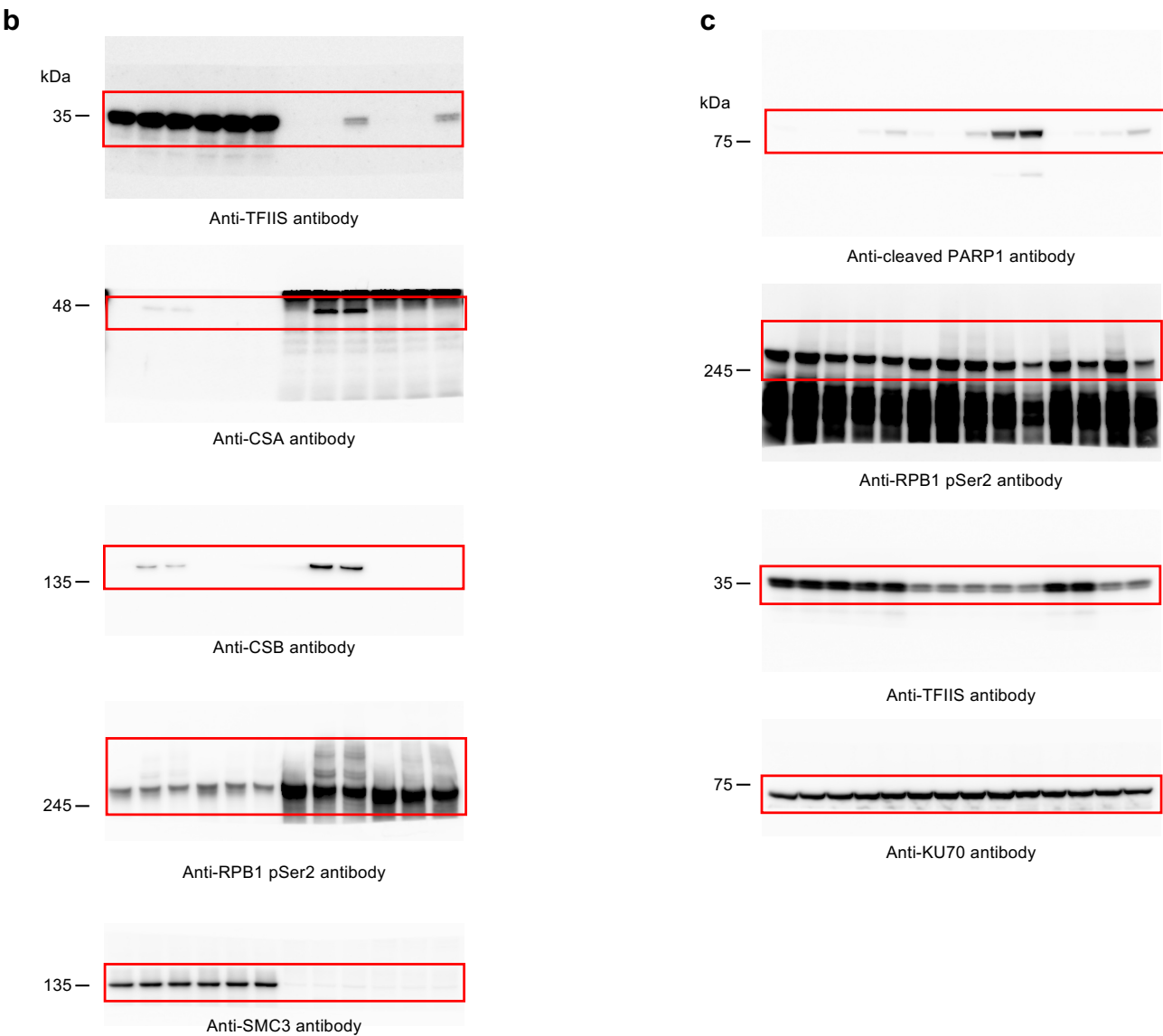

**f**

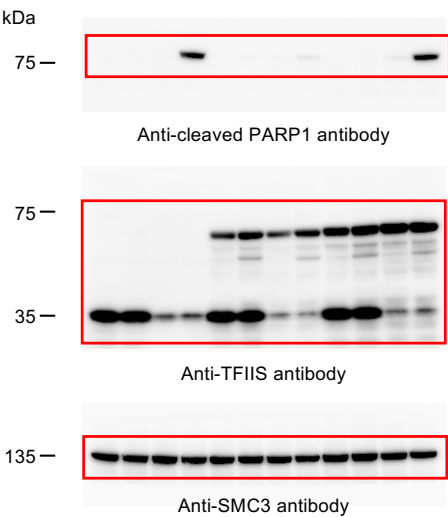

Supplement: Supplementary file 5 — Unprocessed western blots. [file 41556_2024_1401_MOESM5_ESM.pdf]

**d**

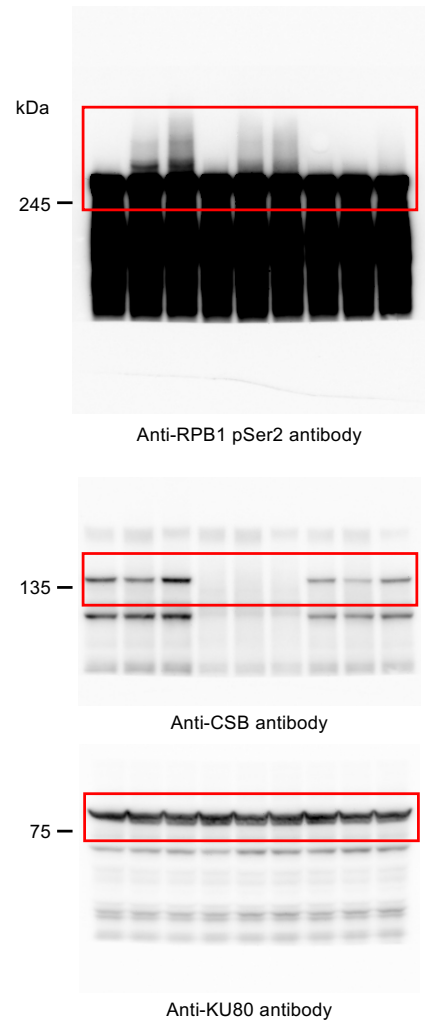

**e**

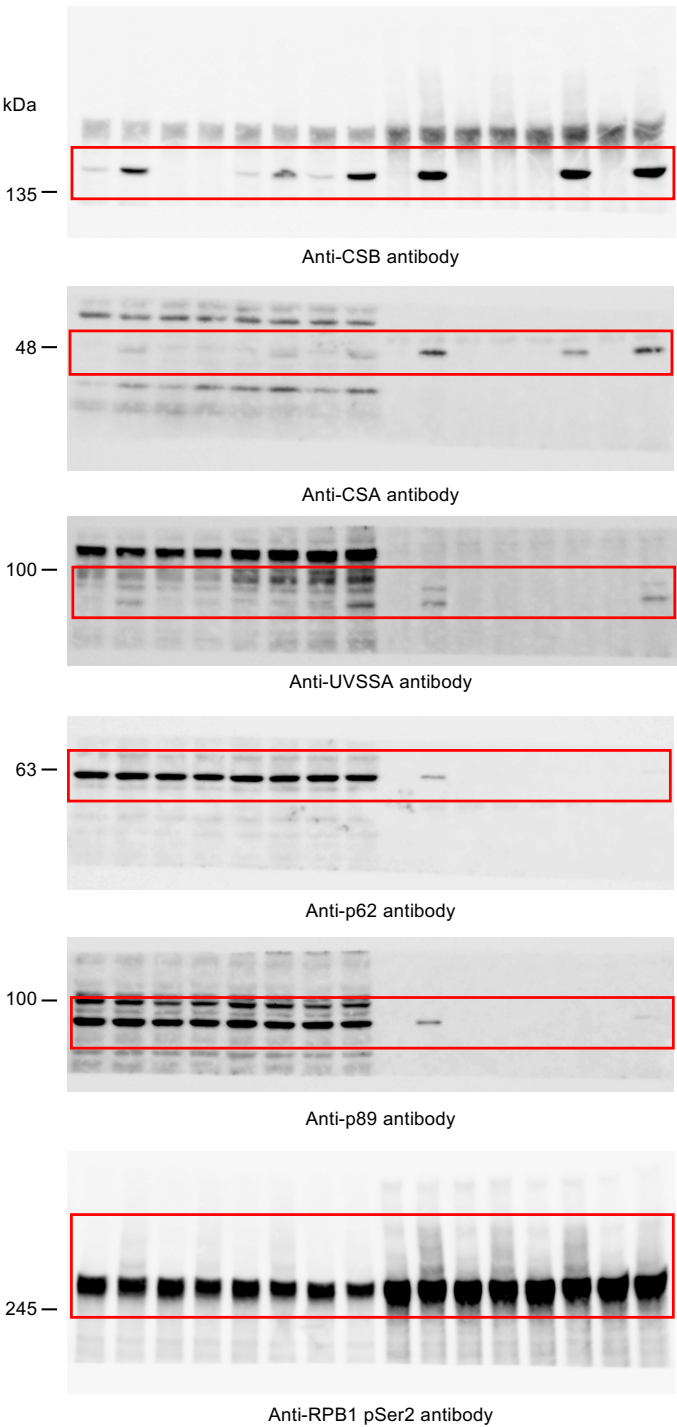

Supplement: Supplementary file 6 — Unprocessed western blots. [file 41556_2024_1401_MOESM6_ESM.pdf]

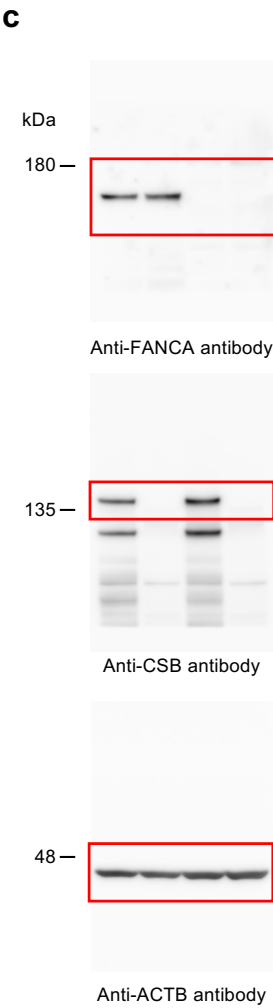

Supplement: Supplementary file 7 — Unprocessed western blots. [file 41556_2024_1401_MOESM7_ESM.pdf]

Extended Data Fig. 4 uncropped images

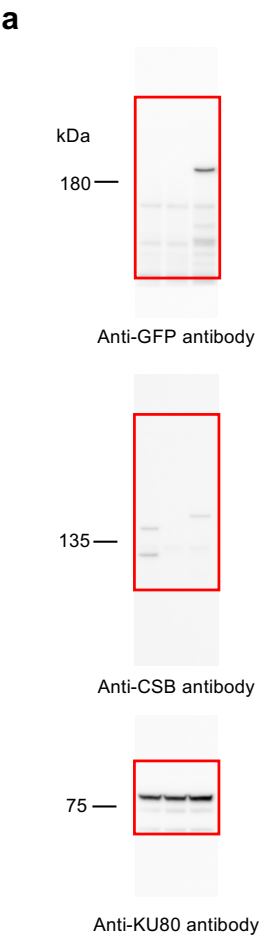

Supplement: Supplementary file 8 — Unprocessed western blots. [file 41556_2024_1401_MOESM8_ESM.pdf]

**a**

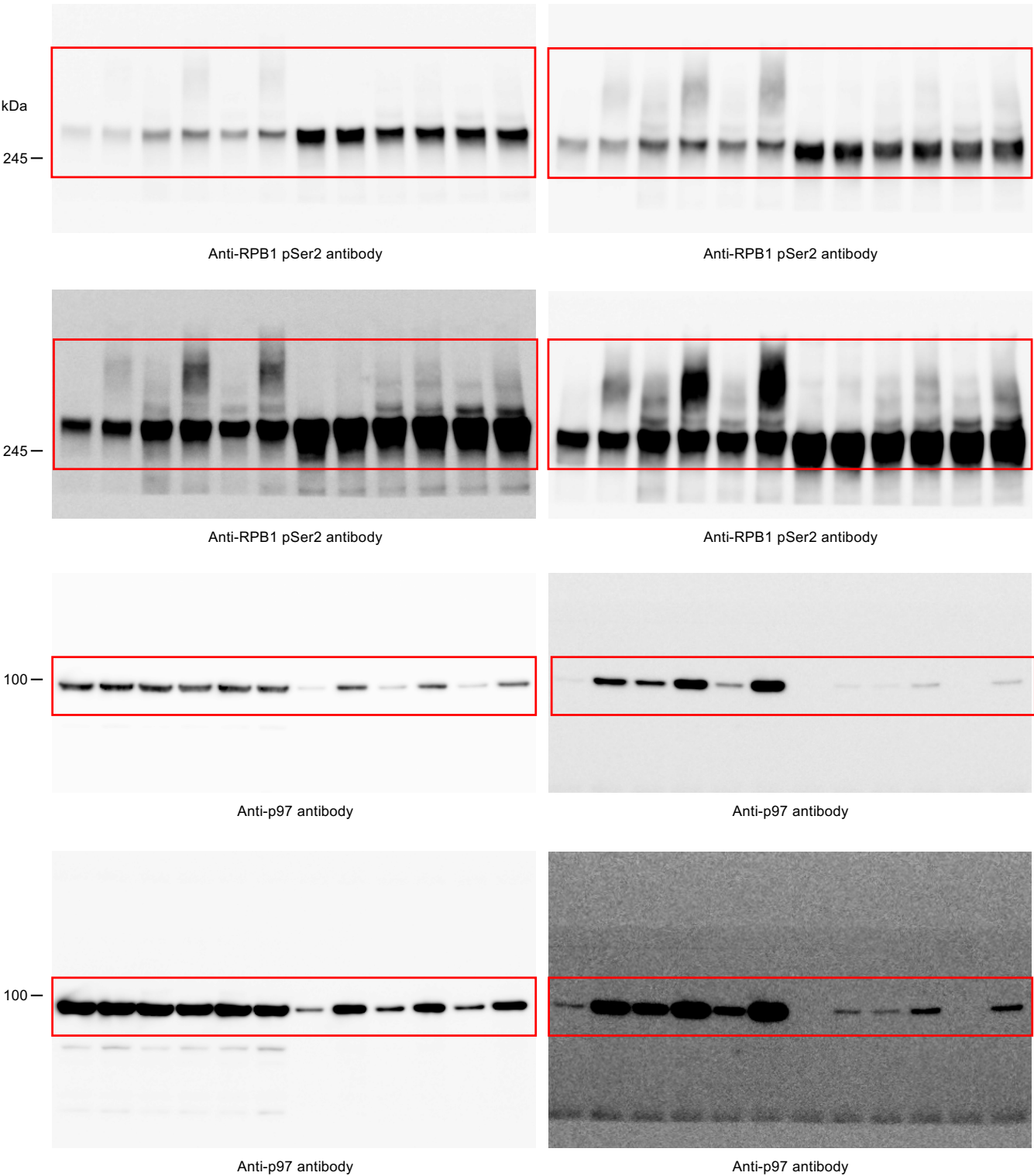

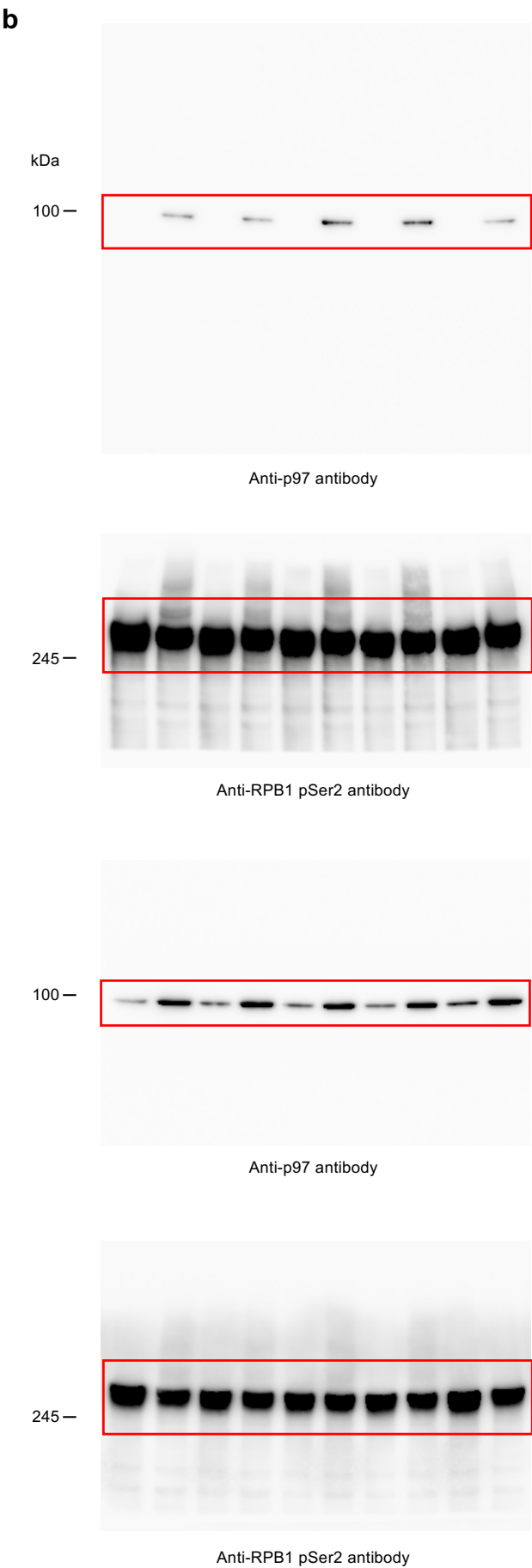

Supplement: Supplementary file 9 — Unprocessed western blots. [file 41556_2024_1401_MOESM9_ESM.pdf]

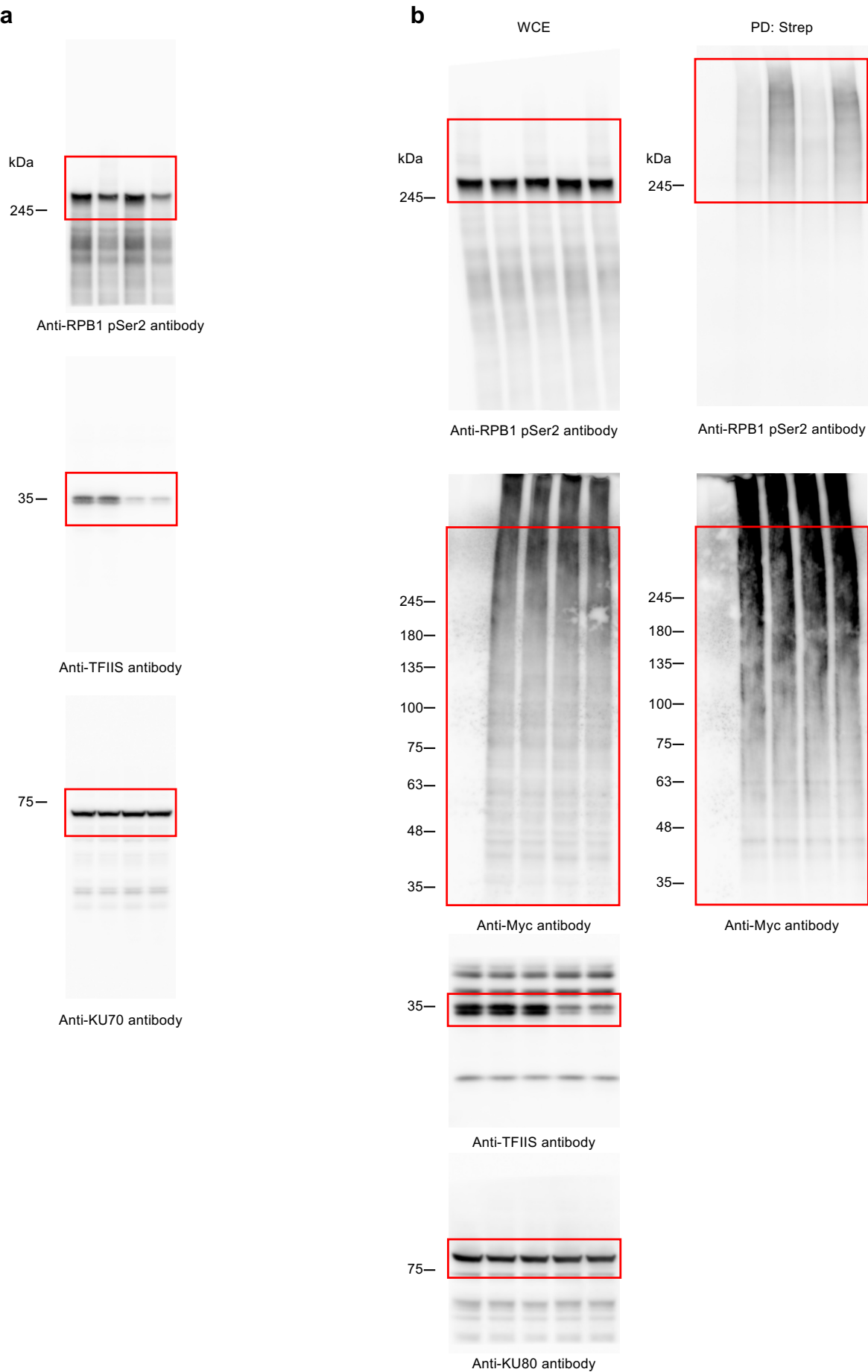

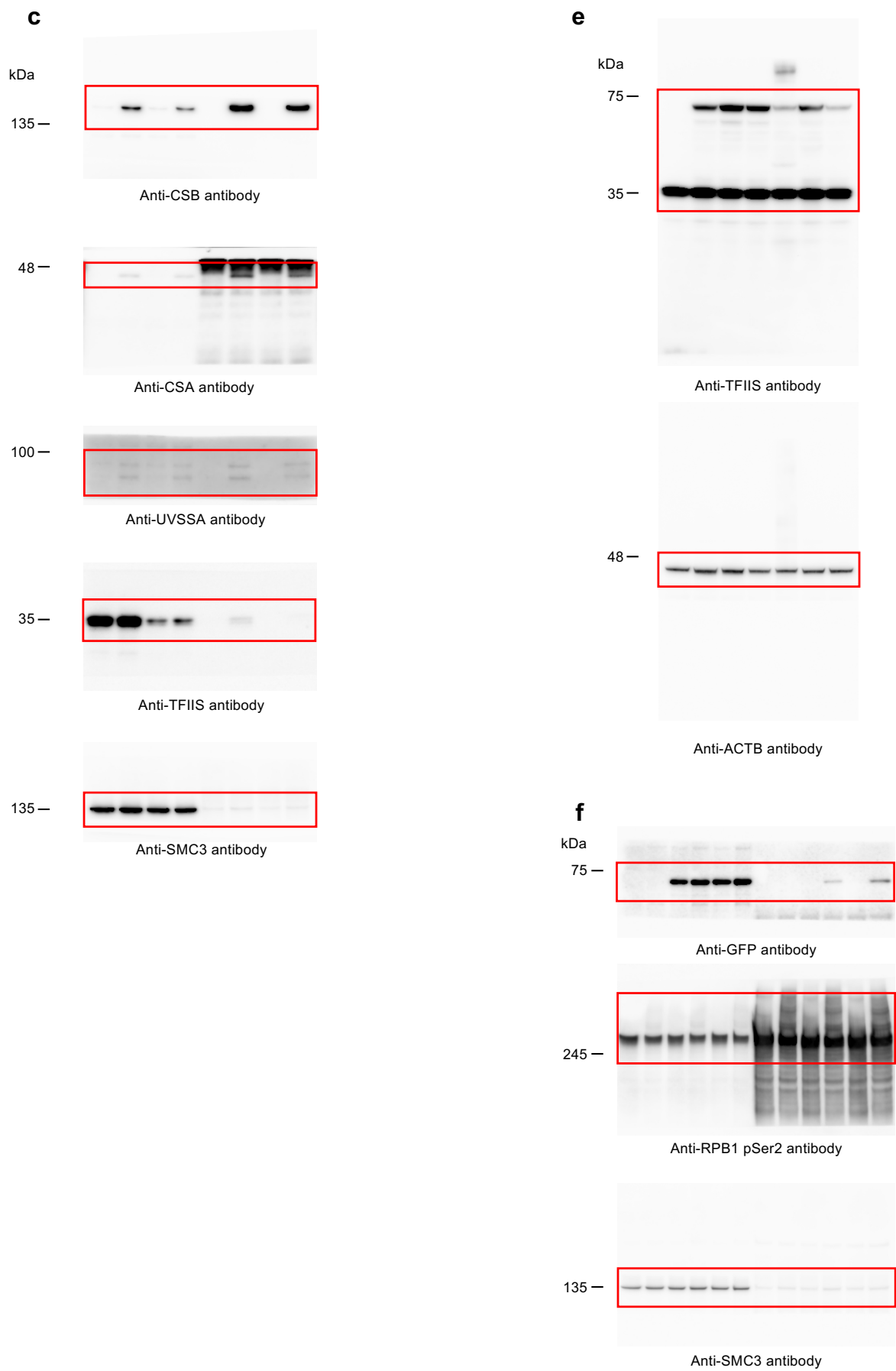

Supplement: Supplementary file 10 — Unprocessed western blots. [file 41556_2024_1401_MOESM10_ESM.pdf]

## Replicate\_uncropped\_ED6f-RPB1

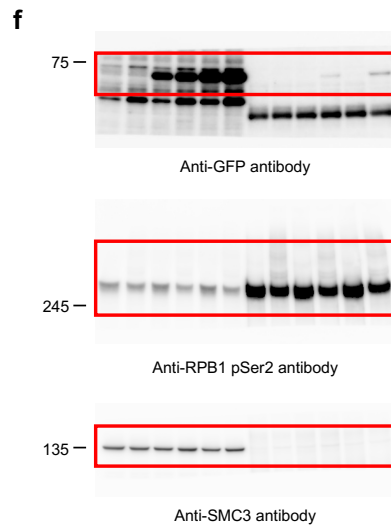

## Replicate\_ED6f-RPB1

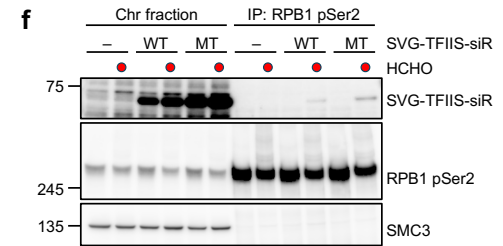

Supplement: Supplementary file 11 — Alternative unprocessed and processed western blots (ED6f-RPB1). [file 41556_2024_1401_MOESM11_ESM.pdf]
